# Composite Nanoarchitectonics with Turmeric-Functionalized Layered Double Hydroxide/Poly(3-hydroxyoctanoate) as Bioactive Coatings for Bone and Nerve Regeneration

**DOI:** 10.1021/acsami.6c04793

**Published:** 2026-04-23

**Authors:** Katarzyna Haraźna, Sonia Bujok, Kamila Lis, Julita Wesołowska, Maciej Bik, Agnieszka M. Tomala, Karina Niziołek, Martina Nevoralová, Justyna Prajsnar, Hynek Beneš, Maciej Guzik, Agnieszka Sobczak-Kupiec

**Affiliations:** † Department of Materials Engineering, Faculty of Materials Engineering and Physics, 49571 Cracow University of Technology, 37 Jana Pawła II Av., Cracow 31-864, Poland; ‡ Jerzy Haber Institute of Catalysis and Surface Chemistry, 132074 Polish Academy of Sciences, Niezapominajek 8, Cracow 30-239, Poland; § Institute of Macromolecular Chemistry, Czech Academy of Sciences, Heyrovského nám. 2, Prague 6 162 00, Czech Republic; ∥ CUT Doctoral School, Department of Materials Engineering, Faculty of Materials Engineering and Physics, Cracow University of Technology, 37 Jana Pawła II Av., Cracow 31-864, Poland; ⊥ Laboratory of Microscopic Imaging, Maj Institute of Pharmacology, Polish Academy of Sciences, CEPHARES, 12 Smętna Street, Cracow 31-343, Poland; # Faculty of Materials Science and Ceramics, 513364 AGH University of Krakow, Kraków 30-059, Poland

**Keywords:** poly(3-hydroxyoctanoate), layered double hydroxides, turmeric, bone tissue engineering, nerve tissue engineering

## Abstract

The limited regenerative capacity of bone and peripheral nerve tissues, together with the insufficient bioactivity and immunomodulatory control of commercially available coating materials, drives the development of multifunctional biomaterials capable of modulating cellular and immune responses. Herein, bacterially derived poly­(3-hydroxyoctanoate) (P­(3HO)) was applied as a biodegradable matrix for nanocomposites incorporating layered double hydroxides (LDHs) and their turmeric (turm)-functionalized counterparts. Nanocomposite films containing 2 wt.% nanofillers were fabricated by solvent casting. Structural and physicochemical analyses confirmed successful functionalization (up to 60 wt.% turm), with a tendency to form microscale agglomerates within the polymer matrix. These agglomerates contributed to heterogeneous surface topography (R_a_ 0.9–7.9 μm) and governed the structure–property relationship in accordance with the Nanoarchitectonics strategy. Mechanical and surface properties were tunable, especially with a reduction in surface free energy of up to 24% for turm-containing nanocomposites. *In vitro* studies performed on mouse preosteoblastic (MC3T3-E1) and mouse neuroblastoma × rat glioma hybrid neuronal (NG108-15) cell lines confirmed a lack of toxicity, with cell viability exceeding 70% under both indirect and direct test conditions. All turm-functionalized materials supported cell differentiation and proliferation. However, the most favorable biological response was observed for P­(3HO)_Zn/Al-turm, which exhibited enhanced neuronal proliferation of NG108-15 cells. Moreover, this system demonstrated robust immunomodulatory activity, inducing TGF-β1 secretion at ∼1787 pg mL^–1^ (comparable to the M2 phenotype) while maintaining controlled MMP-2 levels (∼19.9 pg mL^–1^) for human monocytic-derived macrophages (THP-1). In contrast, Ca/Al-based nanocomposites promoted osteogenic responses in MC3T3-E1 cells but showed lower neuronal proliferation. Importantly, incorporation of nanofillers overcame the intrinsic limitations of neat P­(3HO), enabling neuronal growth and differentiation. These findings demonstrate that turm-functionalized P­(3HO)/LDHs nanocomposites, designed according to the nanoarchitectonics concept, constitute a versatile platform integrating structural tunability and bioactive immunoregulation, opening remarkable perspectives for advanced coatings targeting bone and nerve tissue regeneration.

## Graphical abstract

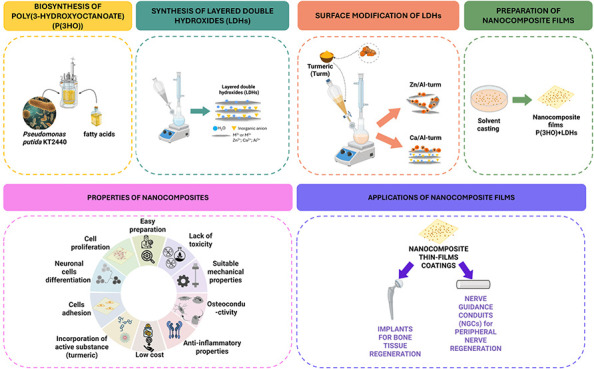

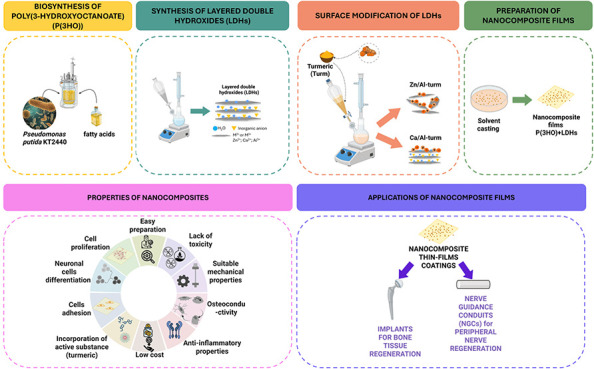

## 1 Introduction

The regeneration of tissues remains a significant clinical challenge, particularly in bone (BTE) and nerve tissue engineering (NTE). Statistical data indicate that the annual incidence of peripheral nerve injury (PNI) in developed countries ranges from 13 to 23 cases per 100,000 individuals.^1^ In parallel, orthopedic surgery frequently requires the use of implants to restore the structural integrity and functionality of damaged bones and joints, with more than two million such procedures performed worldwide each year.^2^ Both peripheral nerve and bone tissues exhibit limited intrinsic regenerative capacity, which often results in incomplete functional recovery after injury.^3^ Autologous grafting remains the clinical gold standard for repairing damaged nerves and bone defects. However, this approach is associated with substantial drawbacks, including donor site morbidity, the need for secondary surgical interventions, and an increased risk of infection and inflammatory complications.^2–6^ Consequently, alternative therapeutic strategies are urgently needed. Tissue engineering (TE) has emerged as a promising approach for the regeneration of damaged tissues and organs. By integrating biomaterials, cells, and bioactive cues, TE strategies aim to develop advanced scaffolds that provide structural support and instructive signals to guide cell behavior and promote functional tissue regeneration.^1,3,7^

In recent years, composites based on polyhydroxyalkanoates (PHAs) have gained increasing attention as materials for medical applications.^8–11^ PHAs are a class of biopolyesters produced by bacterial fermentation and accumulated within microbial cells in the form of intracellular granules. Owing to their biological origin, biodegradability, and biocompatibility, PHAs represent a promising alternative to conventional petroleum-derived polymers. An additional advantage of PHA production is the ability to employ renewable and waste-derived substrates, including byproducts and residues from the agricultural, food, biofuel, and polymer industries, thereby contributing to the development of sustainable and circular material technologies.^11,12^ The biodegradability, resorbability within the human body, lack of cytotoxicity and immunogenicity, as well as the biocompatibility of PHAs are key properties determining their applicability in TE.^8–11,13^ PHAs can be classified into two main groups based on the length of the monomeric units forming the polymer chain: short-chain-length PHAs (scl-PHAs), whose most commonly described representative is the brittle and rigid poly­(3-hydroxybutyrate) P­(3HB), and medium-chain-length PHAs (mcl-PHAs), represented, for example, by the elastic poly­(3-hydroxyoctanoate) P­(3HO).^11,14^ By selecting an appropriate PHA polymer or its blends and adjusting their mass composition, it is possible to obtain polyesters with precisely tailored properties, such as crystallinity, degradation rate, tensile strength, and stiffness (Young’s modulus). Taking these factors into account, specific formulations can be designed for targeted applications in TE, including NTE and BTE.^6^ Control over the degradation rate of PHAs is crucial in the design of drug delivery systems (DDS) with controlled release profiles.^11,14^ The covalent bonding of active substance molecules to PHAs or to oligomers formed during their degradation, *via* ester or amide linkages, represents an effective strategy for achieving controlled release of bioactive compounds. This approach has been widely explored not only in TE applications, such as delivery systems for nonsteroidal anti-inflammatory drugs (NSAIDs), including diclofenac.^8–10^

The ideal scaffold for BTE will exhibit osteoconductive properties and the ability to osseointegrate. The scaffold should provide adequate load-bearing capacity and interact with the extracellular matrix (ECM) to support the healing of damaged bone tissue.^9,11,13,15^ Previous studies have demonstrated that P­(3HO) is a suitable matrix for porous ceramic sponges based on β-tricalcium phosphate (β-TCP), a compound closely resembling natural bone. Consequently, the presence of β-TCP promotes cell adhesion, proliferation, and the induction of new tissue mineralization.^9,13,15^ Cichoń et al. demonstrated that incorporating P­(3HO) as a matrix into β-TCP ceramic scaffolds does not significantly alter compressive strength but markedly improves failure behavior. Although the compressive strength of P­(3HO)/β-TCP composites was comparable to that of pristine β-TCP, the composites exhibited enhanced structural integrity and nonbrittle fracture, which is clinically relevant as it may reduce implant fragmentation and subsequent inflammatory responses.^15^ Moreover, P­(3HO)/β-TCP composites fulfill the mechanical requirements for cancellous bone substitutes, with compressive strength values falling within the range of 1.5–12.0 MPa.^16^ Considering the favorable mechanical properties of P­(3HO), further investigations into the use of P­(3HO)-based nanocomposites as coating materials for implants in BTE are well justified.

PHAs have also been employed in the fabrication of artificial, hollow, cylindrical nerve guidance conduits (NGCs) for NTE. Materials selected for the construction of NGCs must exhibit a carefully tailored combination of physicochemical and mechanical properties, including adequate flexibility, controlled permeability, fluid uptake capacity, and a degradation rate synchronized with the pace of nerve regeneration. Properly designed NGCs provide a biomimetic microenvironment that supports and protects regenerating nerves by promoting directed axonal extension across the injury gap while simultaneously minimizing the detrimental effects of the surrounding tissue environment.^17^ Mcl-PHAs, including P­(3HO), its copolymers such as poly­(3-hydroxyoctanoate-*co*-3-hydroxydecanoate) P­(3HO-*co*-3HD), as well as blends of mcl-PHAs with P­(3HB), have been extensively investigated as materials for the fabrication of NGCs. Owing to their high flexibility, low stiffness, and favorable thermal characteristics, mcl-PHAs exhibit mechanical properties that closely resemble those of native nerve tissue.^17^ This represents a significant advantage over commonly used commercial polymers, including poly­(lactic acid) (PLA), poly­(glycolic acid) (PGA), poly­(lactic-*co*-glycolic acid) (PLGA), and poly­(ε-caprolactone) (PCL), which are typically more rigid and less compliant.^1,6,7,17^ However, it should be noted that PHAs do not exhibit conductive properties, which are essential parameters for NSCs. To give materials made from PHAs additional functionality, i.e., bioactivity, which may be important in nerve regeneration, various inorganic materials are added, such as Bioactive glass 45S5.^7^

Unique features of two-dimensional (2D) nanomaterials, including their chemical versatility, large specific surface area, biocompatibility, and tunable composition, have enabled a wide range of biomedical applications.^5,18–20^ Among these, layered double hydroxides (LDHs) have attracted considerable attention. LDHs can be described by the general formula $[M_{1-x}^{2+}M_{x}^{3+}{\left(OH\right)}_{2}{]}^{x+}{\left(A^{n-}\right)}_{x/n}\times mH_{2}O$ , where *M* ^2+^ represents a divalent cation, *M* ^3+^ a trivalent cation, and *A* ^*n*–^ an interlayer anion.^21^ These compounds are generally considered to exhibit low toxicity. The lamellar structure of LDHs allows the intercalation of bioactive molecules and their sustained release at the target site.^18^ Magnesium/aluminum LDHs (Mg/Al LDHs) have attracted considerable attention as promising carriers for pesticide delivery. In particular, they provide an effective matrix for the development of controlled-release formulations of pretilachlor.^22^ Nanocomposites derived from zinc/aluminum layered double hydroxides (Zn/Al LDHs) can be effectively utilized as adsorbents for the removal of heavy metal ions (i.e., Pb^2+^) from aqueous solutions. Their structural and surface properties enable high adsorption efficiency and make them promising materials for environmental remediation applications.^23^ Moreover, the adaptable chemical composition, favorable physicochemical properties, and biocompatibility of LDHs make them particularly suitable for applications in bone therapy.^5^ The metal ions incorporated in LDHs participate in complex osteogenic processes, enhancing bone regeneration. Notably, calcium/aluminum LDHs (Ca/Al LDHs) have been shown to neutralize the acidic microenvironment associated with osteoporosis through the gradual degradation of the alkaline LDH structure, thereby promoting anti-inflammatory differentiation of bone macrophages.^20^ Similarly, zinc/magnesium LDHs (Zn/Mg LDHs) can inhibit osteoclast activation and maturation, slowing the progression of osteoporosis.^19^

LDHs have also been increasingly investigated as carriers for natural bioactive compounds. The high chemical diversity and multifaceted biological effects of these natural substances make them promising agents for the treatment of various cancers, neurodegenerative diseases, and tissue regeneration. An example of a naturally derived medicinal compound with a long history of use is turmeric *Curcuma longa*). This plant exhibits a wide range of biological activities.^2^ The principal polyphenol extracted from the rhizome of *C. longa*, responsible for the pharmacological effects of turmeric (turm), is curcumin (cur). Cur possesses anticancer, antimicrobial, anti-inflammatory, antioxidant, and antiarthritic properties, which have led to its extensive medical applications.^24^ Studies have demonstrated that cur incorporated into scaffolds for BTE can enhance osteoblast proliferation, differentiation, and mineralization, thereby promoting bone formation. Furthermore, in addition to its effects on bone tissue, cur positively influences nerve regeneration. Scaffolds containing cur have been reported to reduce apoptosis of Schwann cells while promoting their proliferation and migration, supporting the repair of damaged peripheral nerves.^25,26^

In this work, we introduce bacterially derived P­(3HO) as a versatile and sustainable polymer matrix for the rational design of bioactive nanocomposites, engineered at the interface between materials science and regenerative medicine. By incorporating LDHs, namely Ca/Al LDHs (Ca/Al), zinc oxide nanoparticles (ZnO NPs), and their turm-functionalized counterparts (Ca/Al-turm and Zn/Al-turm), we establish a modular strategy for tailoring surface chemistry and biological functionality without compromising material integrity. The resulting nanocomposites were fabricated using a scalable solvent casting (SC) approach and systematically analyzed to elucidate structure–property-function relationships across physicochemical, morphological, structural, and mechanical domains. Their biological performance was evaluated using complementary in vitro models relevant to bone and peripheral nerve engineering, including preosteoblastic, neuronal, and macrophage cell lines (Scheme 1). This study establishes a transferable materials design strategy that integrates biofunctional surface engineering with the prospective application of P­(3HO)-based nanocomposites in biomedical coatings.

**Scheme 1:**
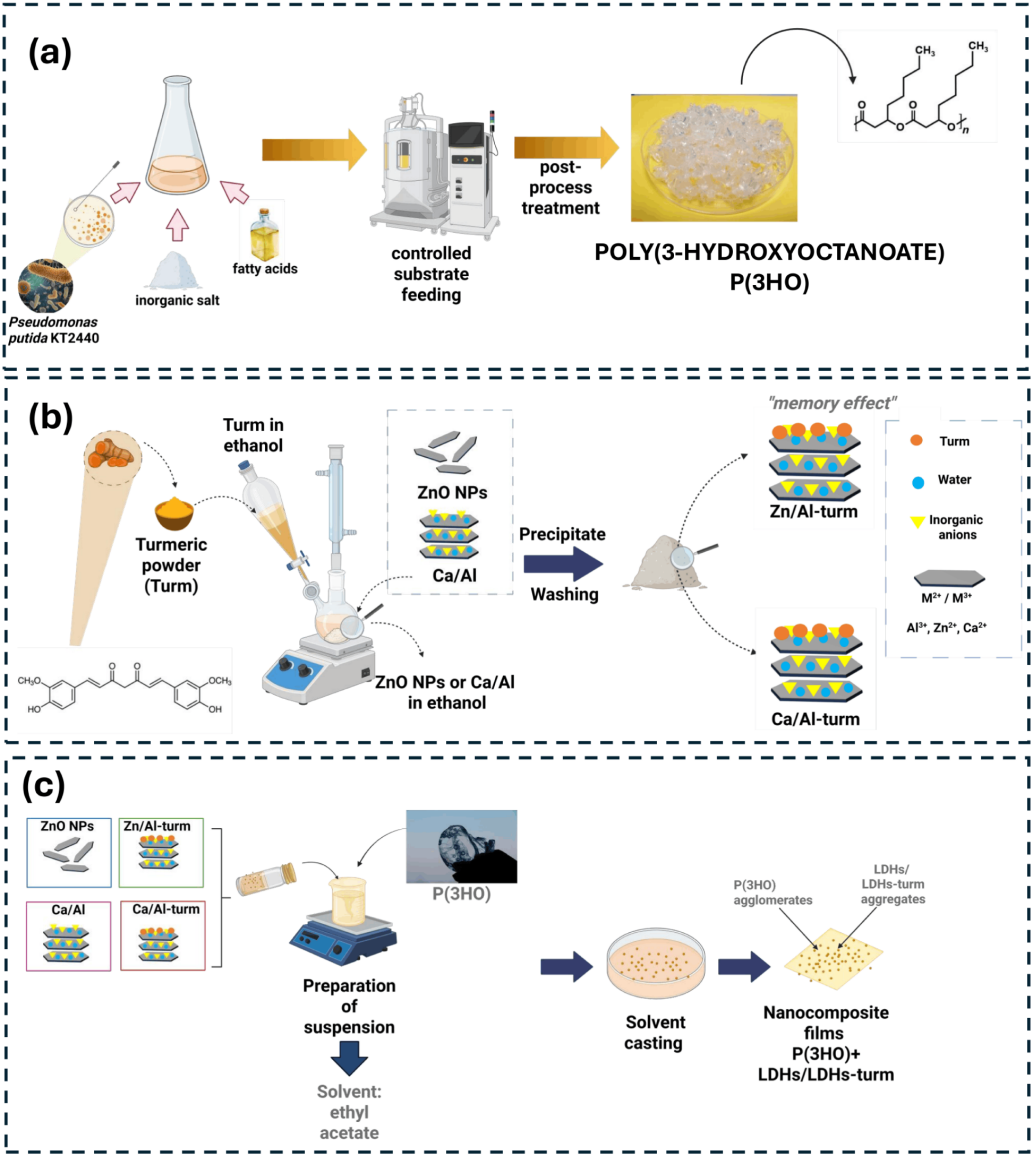
Overview of the Experimental Strategy Employed in This Study, Including **(a)** Biosynthesis of Poly­(3-hydroxyoctanoate) (P­(3HO)), **(b)** Surface Modification of Ca/Al and ZnO NPs: Obtaining of Ca/Al-turm and Zn/Al-turm, and **(c)** Fabrication of P­(3HO)-based Nanocomposite Films^a^

a Created with BioRender.com

## 2 Materials and Methods

### 2.1 Materials

Octanoic acid (≥98%), butyric acid (≥99%), Copper­(II) chloride dihydrate (≥99.95%), Ammonium iron­(III) citrate (16.5–18.5% Fe), Polypropylene glycol P2000, diiodomethane, fetal bovine serum (FBS, sterile-filtered, suitable for cell culture), Penicillin-Streptomycin (10,000 units penicillin and 10 mg streptomycin/mL, 0.1 μm filtered, BioReagent, ideal for cell culture), blue trypan, ethanol 96%, Dullbecco’s phosphate buffered saline (DPBS, tablets), phosphate buffered saline (PBS, tablets), amphotericin B solution (250 μg·mL^–1^ in deionized water, 0.1 μm filtered, BioReagent, suitable for cell culture), l-glutamine (200 mM, BIOXTRA), formaldehyde solution (for molecular biology, 36.5–38.0% in H2O), Dulbecco’s Modified Eagle’s Medium–high glucose (DMEM, 4500 mg/L of glucose), Triton X-100, 0.25% Trypsin in 1 mM EDTA in HBSS, Human TGF-β 1 ELISA Kit, Human MMP-2 ELISA Kit, ethanol, RPMI-1640 medium, 2-mercaptoethanol, resazurin sodium salt, tetramethylrhodamine (TRITC) labeled phalloidin, 4′,6-diamidino-2-phenylindole (DAPI), Lipopolysaccharides from *Escherichia coli* O111:B4 (LPS), Minimum Essential Medium Eagle, Alpha Modification, with sodium bicarbonate and Earl’s salts, without l-glutamine, ribonucleosides, and deoxyribonucleosides (α-MEM), and Interferon-γ Protein, Recombinant human (INF-γ), were procured from Sigma-Aldrich, Darmstadt, Germany. PMA (1 mM) Assay Reagent was obtained from Cayman Chemical, Ann Arbor, MI, USA. Human interleukin-4 (IL-4), and human interleukin-13 (IL-13) were acquired from VWR, Gdańsk, Poland. Hybrid mouse neuroblastoma and rat glioma cells (NG108-15) and Mouse Calvaria-Derived Pre-Osteoblastic (MC3T3-E1) cells were sourced from Sigma-Aldrich, Darmstadt, Germany. The human monocyte cell line (THP-1) was purchased from Cytion, Heidelberg, Germany. Sodium hydroxide, sulfuric acid (96%), sodium phosphate dibasic dodecahydrate, potassium dihydrogen phosphate, ammonium chloride, ammonium sulfate, magnesium sulfate heptahydrate, 20% aqueous ammonia, and sulfuric acid solution 15% were supplied by Chempur, Piekary Śląskie, Poland. Methanol, ethyl acetate, hydrochloric acid 37% vol., calcium chloride, zinc sulfate heptahydrate, manganese­(II) chloride tetrahydrate, nickel­(II) chloride hexahydrate, sodium molybdate dihydrate, and iron­(II) sulfate heptahydrate were delivered by Avantor Performance Materials, Gliwice, Poland. Turmeric was purchased at a local grocery shop. Calcium nitrate tetrahydrate, aluminum nitrate nonahydrate, sodium hydroxide, sodium nitrate, ethanol (analytical grade), methanol (analytical grade), ethyl acetate (analytical grade), and tetrahydrofuran (analytical grade) were obtained from Lach-Ner, Neratovice, Czech Republic. α-Tocopherol (toc), zinc nitrate hexahydrate, and aluminum nitrate nonahydrate were provided by Sigma-Aldrich, Prague, Czech Republic. Urea was supplied by PENTA, Prague, Czech Republic.

### 2.2 Biosynthesis of Poly­(3-hydroxyoctanoate)

The biosynthesis of P­(3HO) was conducted following the methodology described in our previous study^8^. *Pseudomonas putida* KT2440 was employed as the production strain in a 30 L bioreactor. The strain was revived from a −80 °C glycerol stock by streaking onto fresh solid minimal salt medium (MSM) supplemented with sodium octanoate (3.2 g L^–1^) and incubated overnight at 30 °C. A single colony was subsequently transferred to liquid MSM containing sodium octanoate (3.2 g L^–1^) and cultured for 12–15 h. This preculture served as the inoculum for the fermentation at a 1:25 inoculation ratio. An exponential feeding strategy for sodium octanoate was applied in a 30 L fermenter (Biostat B, Sartorius–Stedim, Göttingen, Germany) to enhance P­(3HO) synthesis. Upon completion of fermentation, the biomass was separated from the medium by centrifugation, then frozen and lyophilized. The polymer was dissolved in ethyl acetate, purified with activated carbon, and filtered through a 0.2 μm membrane. Finally, P­(3HO) was precipitated in cold methanol and dried to obtain the purified polymer.

### 2.3 Surface Modification of Nanoparticles Using Turmeric

ZnO NPs and Ca/Al were synthesized following the procedures previously reported.^27^ For surface modification, 0.5 g of Zn/Al or Ca/Al was dispersed in 50 mL of ethanol and stirred under reflux at 60 °C for 1 h. Subsequently, 0.25 g of turm, dissolved in 50 mL of ethanol, was added dropwise to the heated Zn/Al and Ca/Al suspensions. After 24 h, the resulting precipitates were washed with deionized water and subjected to centrifugation three times (20 min, 4000 rpm). The collected solids, designated as Zn/Al-turm and Ca/Al-turm, were dried overnight at 80 °C.

### 2.4 Fabrication of Nanocomposite Films

The solvent casting technique was employed to fabricate the nanocomposite films, as described in our previous work^27^. The synthesized fillers, namely ZnO NPs, Ca/Al, Zn/Al-turm, and Ca/Al-turm, were dispersed in acetone at a concentration corresponding to 2 wt.% of the polymer. The dispersions were subjected to ultrasonic treatment for 30 min, after which 10% w/v P­(3HO) was added, and the mixture was stirred for 1 h. Then, the resulting suspensions were cast into Petri dishes and allowed to dry and condition for 14 days at ambient conditions to ensure complete solvent evaporation. The final nanocomposite films exhibited a uniform thickness of 0.2 mm.

### 2.5 Methods

#### 2.5.1 Fourier-Transform Infrared Spectroscopy

The organic fraction in the modified nanoparticles and the structure of the nanocomposites were examined by ATR-FTIR spectroscopy using a Spectrum 100 FT-IR spectrometer (PerkinElmer, Waltham, MA, USA) equipped with a universal ATR with a diamond crystal. Spectra were recorded over 4000–650 cm^–1^ with 16 scans per spectrum at a resolution of 4 cm^–1^.

#### 2.5.2 X-ray Diffraction

The crystal structures of the samples were analyzed by X-ray diffraction using a high-resolution X’Pert PRO Explorer diffractometer (GNR Analytical Instruments, Novara, Italy) equipped with a one-dimensional silicon detector. CuKα radiation (λ = 1.54 Å) from an X-ray tube operated at 40 kV and 30 mA, monochromatized with a Ni β-filter, was employed. Data were collected over a 2θ range of 1–50° with a step size of 0.1° and an exposure time of 15 s per step.

#### 2.5.3 Raman Spectroscopy

Structural studies of the presented specimens also included Raman spectroscopy. It was used with the WITec Alpha 300M+ spectrometer. A laser of 785 nm excitation wavelength was applied, as well as a grating 300 (ca. 1.3 cm^–1^ spectral resolution), an objective 50× and an Andor CCD detector. Laser power was adjusted using the oscilloscope live view and the WITec TruePower unit to control the power at the level of 70 mW. At least three spectra were recorded for various grains and then averaged in OPUS 7.2 software. Each point measurement included either 10 accumulations with 20 s integration time for each accumulation (ZnO NPs and Ca/Al-LDH) or 800 accumulations with 0.1 integration time for each accumulation (Ca/Al-turm and Zn/Al-turm) in order to reduce the fluorescence effect coming from turm. Spectra acquisition and further postprocessing (cosmic spike removal and cutting the interesting range: 55–3600 cm^–1^) were performed in WITec Project SIX software.

#### 2.5.4 Nitrogen Adsorption and Porosity Analysis

Low-temperature nitrogen adsorption measurements at 77.35 K were carried out using an Autosorb iQ analyzer (Anton Paar, Graz, Austria). Prior to the measurements, the samples were degassed under vacuum using a multistep procedure: heating to 80 °C (2 °C min^–1^, 30 min), followed by 120 °C (2 °C min^–1^, 30 min), and subsequent treatment at 300 °C (5 °C min^–1^, 400 min). Adsorption isotherms were collected over a relative pressure (p/p_0_) range up to 0.89. This limitation of the pressure range enabled selective evaluation of primary (intercrystalline) mesoporosity while reducing the contribution of macroporosity associated with powder agglomeration. The specific surface area (S_BET) was calculated using the Brunauer–Emmett–Teller (BET) model within the relative pressure range of p/p_0_ = 0.05–0.30. Microporosity was evaluated using the t-plot method with the de Boer standard thickness model. The mesopore size distribution was calculated from the adsorption branch of the isotherm using the Barrett–Joyner–Halenda (BJH) method. The textural analysis was further complemented by determination of the surface fractal dimension using the Frenkel–Halsey–Hill (FHH) model. Moreover, calculations based on nonlocal density functional theory (NLDFT) were performed as a complementary approach to classical thermodynamic models. An NLDFT adsorption model with a silica/zeolite kernel and mixed cylindrical–spherical pore geometry was applied, allowing determination of the dominant pore size (mode). All data were analyzed using Anton Paar Kaomi for Nova software (version 2.0).

#### 2.5.5 Scanning Electron Microscopy

The morphology of the nanoparticles and nanocomposites before and after incubation, and the materials following proliferation tests, was examined by scanning electron microscopy (SEM) combined with energy-dispersive spectroscopy (EDS) (JEOL, MA, USA) at an accelerating voltage of 20 kV. Before SEM analysis, a gold nanolayer was deposited on the samples using a DII-29030SCTR Smart Coater sputtering system (JEOL Ltd., MA, USA).

#### 2.5.6 Film Incubation

Nanocomposite films (100 mg) were incubated in 10 mL of Simulated Body Fluids (SBF) and Ringer’s solution (Table S8) in sterile, sealed containers at 36.6 °C for 41 days using a POL-EKO ST 5 B SMART incubator (Wodzisław Śląski, Poland). After incubation, the materials were dried and examined by SEM.

#### 2.5.7 Thermogravimetric Analysis

Thermogravimetric analysis (TGA) of the nanoparticles and nanocomposites was performed using a Pyris 1 thermal analyzer (PerkinElmer, Waltham, MA, USA) under an air atmosphere. Measurements were carried out over a temperature range of 30–800 °C at a heating rate of 10 °C min^–1^, with a purge gas flow rate of 25 mL min^–1^.

#### 2.5.8 Differential Scanning Calorimetry

The thermal properties of the nanocomposites were evaluated by differential scanning calorimetry (DSC) using a Q2000 calorimeter (TA Instruments, New Castle, DE, USA) under a nitrogen purge gas flow of 50 mL min^–1^ in heating–cooling–heating mode. Measurements were performed over −80 °C to 200 °C at a heating rate of 10 °C min^–1^, and the degree of crystallinity was calculated following the method of Haraźna et al.^9^

#### 2.5.9 Tensile Tests

Tensile tests in the elastic region were performed on a DMA850 (TA Instruments, USA) at a 10 mm min^–1^ loading rate with a 0.05 N preload. The dimensions of the specimens were 0.2 × 10 × 20 mm. The Young’s moduli were determined from the linear fitting of the obtained data.

#### 2.5.10 Wettability Measurement

The water and diiodomethane contact angles of P­(3HO) and nanocomposite films were determined using a system goniometer (Advex Instruments, Brno, Czech Republic). For each material, five independent measurements were performed and averaged.

#### 2.5.11 Surface Tension

The Owens-Wendt (OW) method was used to determine the surface tension (*γ_S_*). The polar $\left(\gamma_{S}^{P}\right)$ and dispersive $\left(\gamma_{S}^{D}\right)$ components were calculated using the following equations:

$$\gamma_{S}=\left(\gamma_{S}^{D}\right)+\left(\gamma_{S}^{P}\right)$$

$${\left(\gamma_{S}^{D}\right)}^{0.5}=\frac{\left[\gamma_{d}\left(\cos\theta_{d}+1\right)-\sqrt{\frac{\gamma_{d}^{P}}{\gamma_{w}^{P}}}\gamma_{w}\left(\cos\theta_{w}+1\right)\right]}{2\left[\sqrt{\gamma_{d}^{D}}-\sqrt{\gamma_{d}^{P}\frac{\gamma_{d}^{D}}{\gamma_{w}^{P}}}\right]}$$

$${\left(\gamma_{S}^{D}\right)}^{0.5}=\frac{\left[\gamma_{w}\left(\cos\theta_{w}+1\right)-2\sqrt{\gamma_{S}^{D}\times \gamma_{w}^{D}}\right]}{2\sqrt{\gamma_{w}^{P}}}$$

Where:

$\gamma_{S}^{D}$ – the dispersive component of *γ_S_*

$\gamma_{S}^{P}$ – the polar component of *γ_S_* of investigated sample,

$\gamma_{d}$ – the *γ_S_* of diiodomethane (50.8 mN m^–1^),

$\gamma_{d}^{D}$ – the dispersive component of diiodomethane (48.5 mN m^–1^),

$\gamma_{d}^{P}$ – the polar component of diiodomethane (2.3 mN m^–1^),

$\gamma_{w}$ – the *γ_S_* of water (72.8 mN m^–1^),

$\gamma_{w}^{d}$ – the dispersive component of water (21.8 mN m^–1^),

$\gamma_{w}^{P}$ – the polar component of water (51.0 mN m^–1^),

θ_*d*_– the contact angle of diiodomethane,

θ_*w*_– the contact angle of water.

#### 2.5.12 Topographical Measurements of Surfaces

Optical imaging was performed using a high-precision Keyence VHX-7000 digital microscope (Keyence Corp., Osaka, Japan). Images were acquired in 4K mode with a resolution of 4000 × 3000 pixels. Observations were conducted at magnifications ranging from 20× to 1500× using the high-resolution HDR function, which enabled enhanced visualization of low-contrast features and areas with significant height variations through depth composition. Moreover, the integrated 4K CMOS sensor allowed both two-dimensional and three-dimensional measurements, including surface roughness analysis.

### 2.6 In Vitro Studies on Cell Lines

#### 2.6.1 Maintenance of Cell Lines

NG108-15, MC3T3-E1 Subclone 14 were cultivated in Dulbecco’s Modified Eagle Medium (DMEM) supplemented with a glucose concentration of 4500 mg L^–1^ and α-Minimum Essential Medium (α-MEM), respectively. The culture medium was further enriched with 10% vol. Fetal bovine serum (FBS), 2.5 mM glutamine, and antibiotics, including penicillin (10 μg mL^–1^), streptomycin (100 μg mL^–1^), and amphotericin (0.625 μg mL^–1^). THP-1 cells were maintained in RPMI-1640 medium, which was supplemented with 0.05 mM 2-mercaptoethanol, 10% FBS, 2.5 mM glutamine, and a combination of penicillin at 100 U mL^–1^, streptomycin at 100 μg mL^–1^, and amphotericin at 1.25 μg mL^–1^. The cultures were incubated at 37 °C in a humidified atmosphere with 5% CO_2_ to provide optimal conditions for cell growth and viability. Cells were passaged every 2–3 days, and culturing continued until they reached approximately 85–90% confluence.

#### 2.6.2 Indirect Cytotoxicity Tests

To conduct the resazurin spectrophotometric assay, MC3T3-E1 and NG108-15 cells were seeded into 96-well plates at a density of 2 × 10^4^ cells per well, with each well containing 200 μL of α-MEM and DMEM, respectively, according to ISO 10993-5. The plates were incubated for 24 h at 37 °C in a 5% CO_2_ atmosphere. At the same time, extracts were prepared in accordance with ISO 10993-12 standards. For this process, 100 mg of the appropriate biomaterial was incubated in 1 mL of either α-MEM or DMEM medium for 24 h at 37 °C in a controlled atmosphere with 5% CO_2_. Following this incubation period, the medium was removed and substituted with 200 μL of the previously prepared extracts. The plates were then returned to the incubator for 24 h. For the positive control, 0.1%vol. Triton X-100 was applied to the wells for 15 min, after which the samples were rinsed with DPBS. Wells exposed solely to medium served as the negative control. After the second incubation, any remaining DPBS, medium, and extracts were discarded, and 200 μL of a 100 μM resazurin sodium salt solution was added to each well. The plates were incubated in the dark at 37 °C for 4 h in a 5% CO_2_ atmosphere. Absorbance was measured at a wavelength of 570 and 630 nm using a BioTek 800 TS microplate reader800 TS plate reader (BIO-TEK Instruments, MA, USA). The results were reported as a percentage of cell viability in relation to the negative control, which was cultured in medium on a polystyrene plate. The experiments were performed in triplicate.

#### 2.6.3 Direct Cytotoxicity Tests

Cell adhesion was evaluated using MC3T3-E1 and NG108-15 cells in direct cytotoxicity assays. To this, cells were directly seeded onto presoaked biomaterial samples placed in

48-well plates, with each well containing 0.5 mL of α-MEM or DMEM supplemented with 5 × 10^4^ cells. Control cells (positive and negative controls) were cultured on PS wells. The cells were incubated at 37 °C for 48 h. Following incubation, a positive control was prepared by treating selected wells with 0.1%vol. Triton X-100 for 15 min, after which the samples were rinsed with DPBS. Wells containing medium only served as the negative control. Subsequently, the medium and DPBS were removed, and 200 μL of 100 μM resazurin solution was added to each well. Plates were incubated in the dark at 37 °C for 4 h in a humidified atmosphere containing 5% CO_2_. Absorbance was measured at 570 and 630 nm using a BioTek 800 TS microplate reader (BioTek Instruments, MA, USA). Cell viability was expressed as a percentage relative to the negative control (cells cultured in medium on PS). All experiments were performed in triplicate.

#### 2.6.4 Direct Proliferation Tests

A direct proliferation assay was conducted using resazurin spectrophotometry. MC3T3-E1 and NG108-15 cells, at a density of 1 × 10^4^, were suspended in ∼20 μL of α-MEM and DMEM, respectively, and then seeded onto the nanocomposites in 48-well plates. These samples were incubated for 1 h at 37 °C in a 5% CO_2_ atmosphere. After this initial incubation, the medium volume was increased to 0.5 mL per well, while three wells containing only medium were designated as positive controls. The culture plates were maintained in the incubator (37 °C, 5% CO_2_) for a total of 14 days (MC3T3-E1) and 7 days (NG108-15), with the medium being replaced every two days. Following 48 h of incubation with NG108-15 cells, the existing culture medium was replaced with 0.5 mL of serum-free DMEM, supplemented with 2.5 mM glutamine, and antibiotics, including penicillin (10 μg mL^–1^), streptomycin (100 μg mL^–1^), and amphotericin (0.625 μg mL^–1^). Resazurin spectrophotometric assays were performed on the first, third, seventh, 10th, and 14th days to assess the number of cells on the scaffolds. The procedure for the resazurin spectrophotometric test is outlined in section S1.6.2, titled “*Indirect Cytotoxicity Tests*”. All experiments were performed in triplicate.

#### 2.6.5 Staining the Cytoskeletal Components of Cells

After 14 days of direct proliferation assays, the MC3T3-E1 cells on the scaffolds were dehydrated through incubation in 3.7% formaldehyde. Following this step, the cells were thoroughly washed several times with DPBS before being immersed in 0.1%vol. Triton X-100 for 20 min. After this treatment, the cells were washed multiple times with DPBS. Next, the cells were incubated in the dark with a solution containing tetramethylrhodamine (TRITC) labeled phalloidin, diluted at 1:500 in DPBS, along with 4′,6-diamidino-2-phenylindole (DAPI), diluted at 1:1000 in DPBS, for 30 min. After incubation, the materials were washed several more times with DPBS.

#### 2.6.6 Confocal Microscope

Confocal fluorescence imaging was performed using a Leica TCS SP8 WLL confocal laser scanning microscope (Leica Microsystems) equipped with a 10× objective lens. To minimize spectral crosstalk, images were acquired in sequential scanning mode, with DAPI-stained nuclei recorded in the blue channel and tetramethylrhodamine (TRITC)-labeled phalloidin-stained cytoskeletal structures recorded in the red channel. Excitation/emission wavelengths were set to 405/415–470 nm for DAPI and 577/587–650 nm for TRITC, using a PMT and HyD detectors. The pixel size was 568 × 568 nm, corresponding to a field of view of 1162.5 × 1162.5 μm per image. Line averaging (three scans) was applied during acquisition to improve the signal-to-background ratio.

#### 2.6.7 Examination of Cell Morphology on Nanocomposite Films

Following direct proliferation assays performed on days 7 and 14, the cells were fixed by immersing the scaffolds twice in a 4% (v/v) formaldehyde solution. For detailed observation, the cells adhered to the scaffold surfaces were gradually dehydrated by sequential immersion of the scaffolds in ethanol solutions of increasing concentrations (35%, 50%, 70%, and 90%) for 15 min at each step. The dehydration process was completed by two immersions in 96% ethanol. Cell adhesion and proliferation were evaluated using scanning electron microscopy (SEM) with a JEOL IT200 microscope (JEOL Ltd., Tokyo, Japan). Prior to analysis, the scaffolds were sputter-coated with gold using a DII-29030SCTR Smart Coater (JEOL Ltd., MA, USA).

#### 2.6.8 Macrophage Polarization and Characterization

Macrophages were differentiated from THP-1 through stimulation with PMA. The THP-1 cells were seeded into a 24-well plate at a density of 1 × 10^6^ cells per well in 500 μL of basal culture medium (RPMI-1640) supplemented with 200 nM PMA. The introduction of PMA led to the differentiation of monocytes into adherent macrophages. After 1 day of culture, adherent THP-1-derived macrophages, designated as nonactivated macrophages (M0 phenotype), were polarized into M1 and M2 phenotypes. This polarization was achieved through a three-day exposure to specific treatments: (1) The culture medium containing 100 ng mL^–1^ of LPS and 20 ng mL^–1^ INF-γ, (2) while the M2 phenotype was induced with basal medium containing 40 ng mL^–1^ of IL-4 and 20 ng mL^–1^ of IL-13. Macrophages cultured on the biomaterial were maintained in basal medium to assess the biomaterial’s influence on their polarization. Following the polarization phase, all culture media were replaced with fresh basal medium without additional factors, and the macrophages were allowed to culture for a further 7 days. Every 2 days, the culture medium was replaced with a fresh one. The levels of the MMP-2 and the anti-inflammatory cytokine TGF-β1 were assessed after 7 days of macrophage culture. Macrophages were cultured on biomaterials, while reference M0, M1, and M2 cells were maintained in polystyrene wells. The concentrations of MMP-2 and TGF-β1 in the cell culture supernatants were evaluated using commercially available human-specific ELISAs, in accordance with the manufacturer’s protocol.

### 2.7 Statistical Analysis

ANOVA was employed as a one-way analysis of variance to delineate statistically significant differences between the two data sets. The differences observed were considered statistically significant at the following probability thresholds: *p < 0.05, **p < 0.01, and ***p < 0.001. This analysis was performed using Origin Pro 2019 software from OriginLab, MA, USA.

## 3 Results and Discussion

### 3.1 Structural and Morphological Evolution of Turmeric-Modified LDHs

For Zn/Al-turm and Ca/Al-turm, compared to ZnO NPs and Ca/Al (Figure 1c and d), changes in the crystal structure of nanomaterials were observed. First, in our previously published work, we presented diffractograms of unmodified ZnO NPs, which confirmed the structure of calcined ZnO (2θ = 34.0° (002)). After thermal treatment at ≥500 °C, the material contains zinc and aluminum oxides.^27^ Moreover, the crystalline structure of Zn/Al-turm was regenerated, which is due to its ability to restore its original Zn/Al layered structure in aqueous solutions.^28^ Surface modification was also confirmed by the presence of characteristic absorption bands ∼1360 cm^–1^, corresponding to the presence of CO_3_^2–^ (Figure 1a and b).^27^ In turn, the Zn/Al-turm (Figure 1c) diffractogram shows sharp reflections at 2θ: 11.8° (003); 23.6° (006); 34.7° (012); 39.3° (015); 46.9° (018), identical to those obtained after modification of Zn/Al using α-tocopherol.^27^ Similar observations were reported by Vignesh et al. for Ag-SnO_2_ after modification with cur.^29^

**Figure 1:**
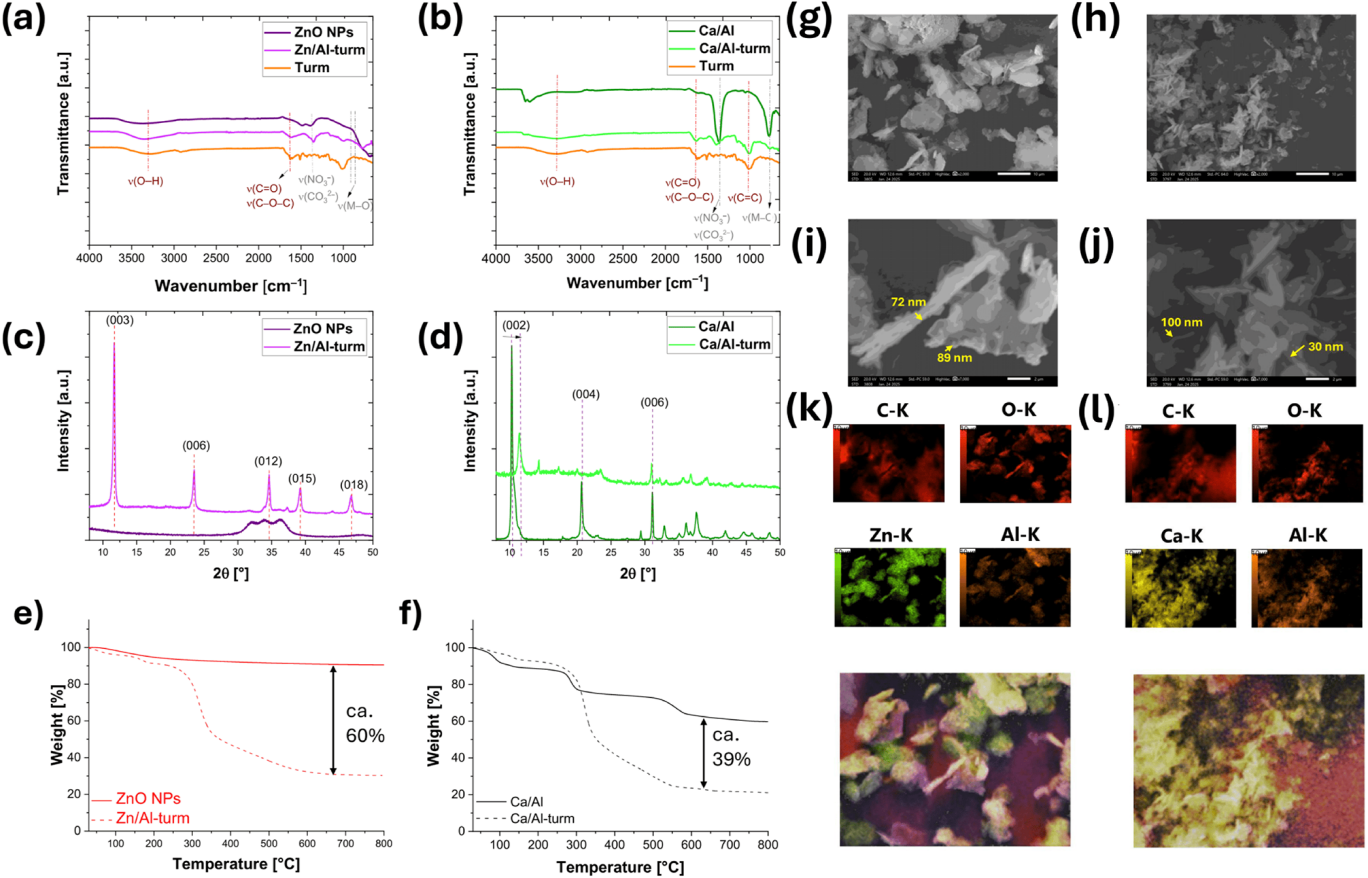
**(a)** IR spectra and **(c)** XRD patterns of ZnO NPs and Zn/Al-turm; **(b)** IR spectra and **(d)** XRD patterns of Ca/Al and Ca/Al-turm. TGA curves of **(e)** pristine ZnO NPs and Zn/Al-turm, and **(f)** pristine Ca/Al and Ca/Al-turm, indicating the amount of turm adsorbed on the material surface, which was 60 wt.% for Zn/Al-turm **(e)** and 39 wt.% for Ca/Al-turm **(f).** SEM micrographs with corresponding EDX elemental maps of Zn/Al-turm **(g, i, k)** and Ca/Al-turm **(h, j, l)**; the highlighted dimensions in panels **(i)** and **(j)** confirm that the synthesized nanostructures exhibit particle sizes below 100 nm.

In the case of the Ca/Al-turm diffractogram, a shift of the peak corresponding to the (002) plane was observed compared to Ca/Al, from 10.3° to 11.7° of 2θ (Figure 1d). Moreover, the Ca/Al-turm diffractogram lacks the peak associated with the (004) plane, which was present in the Ca/Al sample. Furthermore, after modification, a decrease in the basal spacing of Ca/Al-turm was noted (Table S2). This effect may be attributed to the adsorption of turm onto the LDH surface, driven by strong interactions between the phenolic groups of turm and the hydroxyl groups of the LDH layers. A similar phenomenon was reported by Madhusha et al. during the surface modification of Mg/Al-LDH with cur.^30^ Furthermore, consistent with our previous findings, no shift of the (003) reflection from its characteristic position associated with carbonate anion intercalation was observed (2θ = 11.8°, d = 0.75 nm; Figure 1d).^27^ This may also be related to the exchange of anions between layers, i.e., NO_3_^–^ for CO_3_^2–^, as observed previously by Beneš et al.^31^

TGA analysis enabled the determination of the weight percentage of the organic compound adsorbed on the nanoparticle surface. For Zn/Al-turm and Ca/Al-turm, adsorption levels of 60 wt.% and 39 wt.% were obtained, respectively (Figure 1e and f). Compared with our previous study, in which analogous nanoparticles were modified with α-tocopherol (Zn/Al-toc and Ca/Al-toc), significantly lower loadings of the active compound were observed, at 8 wt.% and 13 wt.% levels, respectively.^27^ In that case, only minor changes in the characteristic absorption bands of LDH were detected in the FTIR and Raman analyses (Figure S1; for additional details, please see Raman Analysis in the *Supporting Information: S1.1 Raman analysis of LDHs*). In contrast, in the present study, distinct absorption bands corresponding to characteristic vibrational modes of turm functional groups were clearly observed in the FTIR spectra of Ca/Al-turm and Zn/Al-turm, including ν­(O–H) (∼3300 cm^–1^), ν­(C=O) and ν­(−C–O–C) (∼1630 cm^–1^), as well as ν­(C=C) vibrations in aromatic rings (∼1550 and 1510 cm^–1^).^2,3^ However, it should be noted that NO_3_^–^ and CO_3_^2–^ absorption bands are visible in the range from 1360 to 1410 cm^–1^, which is consistent with XRD diffractograms that revealed the presence of intercalated nitrate/carbonate anions, while analyses confirmed surface modification by turm.^27,32^ Zn/Al-turm (Figure 1g and i) and Ca/Al-turm (Figure 1h and j) were characterized morphologically using SEM, which allowed the dimensions of the nanomaterials to be assessed: in the smallest dimension, their size was less than 100 nm, while in the other, it was several μm. Then, EDX analysis confirmed the elemental composition of both Zn/Al-turm (Figure 1k) and Ca/Al-turm (Figure 1l). Moreover, the Table S1 presents the textural properties of the obtained LDHs, including specific surface area, pore volume and size, as well as structural parameters determined using various analytical methods, i.e., BET, t-plot method (de Boer), Barrett–Joyner–Halenda (BJH), Non-Local Density Functional Theory (NLDFT), and Frenkel–Halsey–Hill (FHH). Additional details are provided in the *Supporting Information: S1.2 BET analysis of obtained powders*. Notably, in the case of ZnO NPs, a dominant mesopore diameter of 15.7 nm and a fractal dimension (D_s_) of 2.4 were observed. Such surface roughness, combined with an accessible mesoporous network, suggests a high availability of active sites, which may also indicate potential applicability of these materials in surface-related processes (e.g., photocatalysis).^21^ Importantly, for all analyzed materials, relatively high D_s_ values in the range 2.4–2.6 were recorded (Table S1), indicating their potential applicability in adsorption processes.^23^

### 3.2 Structure–Property Relationships of P­(3HO)-Based Nanocomposites

The IR spectra of nanocomposites containing Zn/Al-turm (Figure S2) and Ca/Al-turm (Figure S3) predominantly exhibit absorption bands characteristic of P­(3HO) functional groups. This is evidenced by the presence of bands assigned to ν­(O–H) stretching vibrations (∼3400 cm^–1^), ν­(C=O) stretching vibrations (1730–1740 cm^–1^), and ν­(C–H) stretching vibrations (∼2925 cm^–1^ and ∼2850 cm^–1^).^27^ This can be attributed to the significant predominance of the polymer matrix relative to the low filler content, which did not exceed 2 wt.%. Nevertheless, the spectra also revealed signals indicating of the presence of the inorganic phase within the composite structure. For materials containing Zn/Al-turm and Ca/Al-turm, bands corresponding to ν­(C=O) and −C–O–C (∼1630 cm^–1^) of turm were observed. Moreover, for Zn/Al-turm, a broad ν­(O–H) band (3650–3150 cm^–1^) of turm was detected. These findings are consistent with XRD analysis, which indicated that the incorporation of Ca/Al-turm and Zn/Al-turm nanoparticles did not result in intercalation with P­(3HO) chains or exfoliation of the fillers. The diffractograms of P­(3HO)_Zn/Al-turm (Figure S4) and P­(3HO)_Ca/Al-turm (Figure S5) displayed reflections originating from both the P­(3HO) matrix and the Zn/Al-turm or Ca/Al-turm nanoparticles. For neat P­(3HO), the diffraction peaks were observed at 2θ = 17.8° (120), 19.4° (140), and 21.6° (031).^27^ The intensity of diffraction reflections originating from the filler in the polymer matrix has previously been linked to the degree of particle dispersion and the absence of exfoliation/intercalation in the P­(3HO) matrix.^27^ For P­(3HO)_Zn/Al-turm, characteristic reflections were observed: 11.8° (003); 23.6° (006). Less intense diffraction peaks originating from nanoparticles were observed for P­(3HO)_Ca/Al-turm: 11.5° (002).

These observations are consistent with the microscopic analyses performed for nanocomposite films containing both unmodified fillers (P­(3HO)_ZnO NPs and P­(3HO)_Ca/Al) and their modified counterparts (P­(3HO)_Zn/Al-turm and P­(3HO)_Ca/Al-turm). SEM and SEM–EDX analyses of P­(3HO) (Figure 2a), P­(3HO)_Zn/Al-turm (Figure 2b), and P­(3HO)_Ca/Al-turm (Figure 2c) demonstrated that, after 41 days of incubation in both SBF and Ringer’s solution (Figure S6), the elemental composition of the films remained unchanged. Moreover, micrographs acquired after 41 days of incubation in SBF and Ringer’s solution revealed microcracks and holes (marked with green arrows), indicating progressive biodegradation of the films (Figure S7). Both before and after incubation, agglomerates originating from ZnO NPs, Ca/Al, Zn/Al-turm, and Ca/Al-turm were observed (indicated by yellow arrows). Furthermore, P­(3HO) agglomerates were observed together with particles, particularly for P­(3HO)_Ca/Al and P­(3HO)_Ca/Al-turm (Figure 2d). Moreover, 3D surface topography analysis revealed that films produced using the SC technique exhibited thickness heterogeneity. The surface displayed local depressions and indentations, which do not correspond to pore structures but rather result from uneven material shrinkage during solvent evaporation and film drying (Figure 2e). This is an important observation because it is related to the formation of micro/nanoroughness, which is important for materials used in tissue engineering, as it significantly affects cellular interactions in both *in vitro* and *in vivo*. Particularly important parameters include average roughness (*R*_*a*_), root-mean-square roughness (*R*_*q*_), and skewness (*R*_*sk*_), as they influence the modulation of cell adhesion, differentiation, and proliferation. This effect arises from the sensitivity of the cellular microenvironment to physical cues transmitted by the implant surface.^33^ The *R*_*a*_ and *R*_*q*_ values obtained for the P­(3HO) and P­(3HO)_Zn/Al-turm samples are comparable. In contrast, statistically significant differences in both roughness parameters were observed for the remaining compositions. Specifically, the *R*_*a*_ and *R*_*q*_ values amounted to 6.5 ± 1.0 and 8.6 ± 3.2 μm for P­(3HO), 2.7 ± 0.9 and 3.4 ± 1.0 μm for P­(3HO)_ZnO NPs, 0.8 ± 0.1 and 1.1 ± 0.2 μm for P­(3HO)_Ca/Al, and 1.0 ± 0.3 and 1.3 ± 0.3 μm for P­(3HO)_Ca/Al-turm, respectively (Table 1). No statistically significant differences were found in the skewness parameter (*R*_*sk*_) between the neat P­(3HO) and the composite samples. Nevertheless, the *R*_*sk*_ indicates that the surfaces of P­(3HO), P­(3HO)_Zn/Al-turm, and P­(3HO)_Ca/Al are dominated by indentation-like features, whereas P­(3HO)_ZnO NPs and P­(3HO)_Ca/Al-turm exhibit pore-like concavities, as evidenced by the negative *R*_*sk*_. Consistent with these observations, Taylor et al. reported that *R*_*a*_ and *R*_*q*_ for PHA blends, such as 50P­(3HB)/50P­(3HO), were more than an order of magnitude higher than those measured for PCL. Moreover, an increase in protein adsorption was correlated with higher *R*_*a*_ and *R*_*q*_, highlighting surface roughness as a key factor promoting enhanced cell adhesion on biomaterial surfaces.^6^ Furthermore, Basnett et al. observed that higher *R*_*a*_ and *R*_*q*_ are correlated with the presence of material heterogeneity, microcracks, and pores.^34^ This is consistent with our results. The *R*_*a*_ and *R*_*q*_ that we obtained are at least 10-fold higher than those obtained by Taylor et al. for 50P­(3HB)/50P­(3HO) (*R*_*a*_: 125.93 ± 55.81 nm; *R*_*q*_: 154.59 ± 62.61 nm).^6^ The micrographs we recorded show pores, cracks, particle agglomerates, and P­(3HO) (Figure 2d and e). According to Basnett et al., an increase in roughness may be associated with the presence of local polymer agglomerates in the case of miscibility of two different polymers characterized by different hydrophobicity.^34^

**Figure 2:**
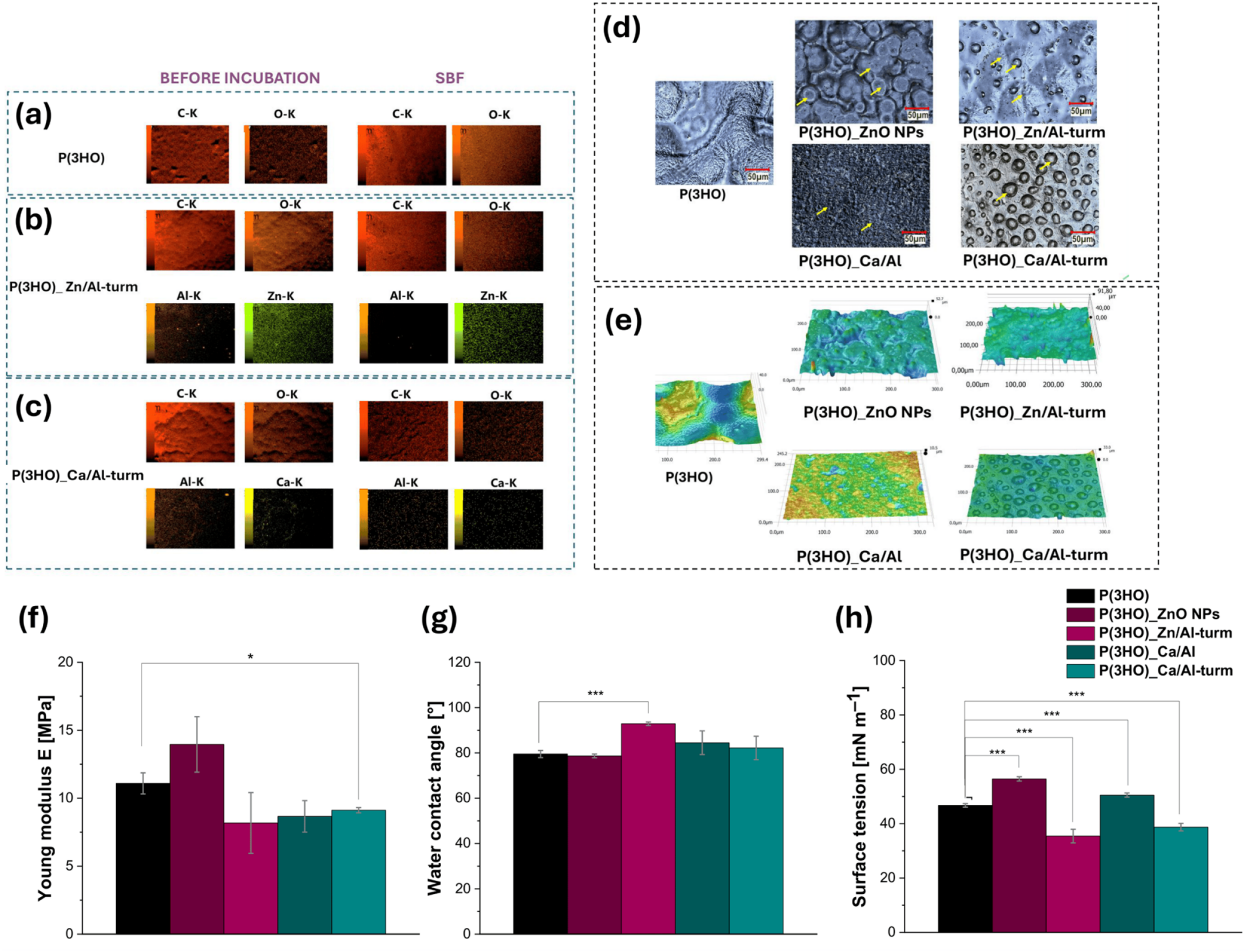
SEM micrographs with corresponding EDX elemental maps of **(a)** P­(3HO), **(b)** P­(3HO)/Zn/Al-turm, and **(c)** P­(3HO)_Ca/Al-turm before incubation and after 41 days of incubation in SBF. The EDX maps confirm the elemental composition of the materials, indicating the presence of C, O, Al, Zn, and Ca, and demonstrate that the functionalized nanoparticles are not leached out during incubation in SBF. **(d)** Optical microscopy images showing the microstructure of nanocomposite films: P­(3HO), P­(3HO)_ZnO NPs, P­(3HO)_Zn/Al-turm, P­(3HO)_Ca/Al, and P­(3HO)_Ca/Al-turm. The yellow arrows indicate agglomerates of nanoparticles. **(e)** Surface profiles of the investigated nanocomposite films revealed nonuniform thickness and surface structure; these profiles were used to determine the films’ surface roughness. **(f)** Young’s modulus (E) of the nanocomposite films (*n* = 5). **(g)** Water contact angle values of the nanocomposite films (*n* = 5). **(h)** Surface tension values of the prepared nanocomposite films (*n* = 30). Data are presented as mean ± SE. Statistical significance was defined as *p < 0.05 and ***p < 0.001.

**Table 1:** Surface Roughness Parameters (*R*_*a*_, *R*_*q*_, and *R*_*sk*_) and Thermal Properties (*T*_*g*_, *T*_*m*_, *T*_*onset*_, and *X*_*c*_) of Pristine P­(3HO) and the Prepared Polymer Nanocomposites^a^

Sample abbreviations	*R*_*a*_[μm]	*R*_*q*_[μm]	*R*_*sk*_[μm]	*T*_*g*_ [^o^C]	*T*_*m*_ [^o^C]	*T*_*d5%*_ [^o^C]	*X*_*c*_ [%]
P(3HO)	6.5 ± 1.0	8.6 ± 3.2	0.9 ± 1.6	–37.4	54.5	248	9.8 ± 0.3
P(3HO)_ZnO NPs	2.7 ± 0.9***	3.4 ± 1.0**	-0.7 ± 0.8	–37.2	52.9	253	10.2 ± 0.8
P(3HO)_Zn/1Al–turm	7.9 ± 1.5	9.9 ± 1.9	0.0 ± 1.0	–50.9/–40.3	53.9	253	11.1 ± 0.8
P(3HO)_Ca/Al	0.8 ± 0.1***	1.1 ± 0.2***	2.3 ± 1.7	–40.7	53.7	236	14.3 ± 0.3
P(3HO)_Ca/Al–turm	1.0 ± 0.3***	1.3 ± 0.3***	-0.6 ± 1.2	–41.7	55.5	246	14.4 ± 0.2

a Surface Roughness Data (*R_a_*, *R_q_*, and *R_sk_*) Were Analysed Using One-Way Analysis of Variance (ANOVA), and Statistically Significant Differences are Indicated as **p < 0.01, and *** p < 0.001. Data are Presented as Mean ± SE. The Thermal Parameters *T_g_*, *T_onset_* and *X_c_* Have Also Been Reported in Our Earlier publication.^27^

Young’s modulus (*E*), a key descriptor of material stiffness, is a critical parameter governing cell-substrate interactions during tissue regeneration. The mechanical properties of biomaterials regulate fundamental cellular processes, including proliferation, migration, and differentiation.^35^ Materials with relatively low stiffness are particularly advantageous in BTE and NTE, as they more closely mimic the mechanical characteristics of the native ECM and provide cells with appropriate mechanical cues. For instance, conventional metallic implants exhibit stiffness values far exceeding those of bone tissue, which can induce stress shielding and impair physiological bone remodeling, consistent with Wolff’s law.^36^ Similarly, excessively stiff substrates may inhibit axonal growth and nerve signal transmission, underscoring the importance of tailoring the mechanical properties of biomaterials to the target tissue.^37^

Tensile tests revealed that the prepared nanocomposite films exhibited lower *E* compared to pristine P­(3HO) for P­(3HO)_Zn/Al-turm, P­(3HO)_Ca/Al, and P­(3HO)_Ca/Al-turm, with reductions of 26%, 22%, and 18% (*p* < 0.05), respectively (Figure 2f and Table S3). In contrast, a moderate increase in *E* (26%) was observed for the P­(3HO)_ZnO NPs nanocomposite, which can be attributed to the homogeneous dispersion of ZnO nanoparticles and the absence of extended defect regions, as evidenced in Figure 2c.^38^ Notably, Figure 2e indicates the presence of P­(3HO) agglomerates in the P­(3HO)_Zn/Al-turm and P­(3HO)_Ca/Al-turm nanocomposites. Such surface defects are known to reduce *E*. Similar trends were reported by Shiv et al., who associated the decrease in *E* with the formation of particles agglomerates that restrict polymer chain mobility and limit reinforcement efficiency at low nanoparticle loadings.^39^ Moreover, nanoparticle agglomeration adversely affects mechanical performance by weakening interfacial interactions. Zare et al. demonstrated, based on the Kerner and Paul models, that uniform nanoparticle dispersion within polymer matrices enhances *E*, whereas nanoparticle agglomeration leads to a reduction of *E*.^40^ Significantly, *E* obtained in this study, particularly for the turm-functionalized nanocomposites, fall within a range suitable for peripheral nerve regeneration. Reported *E* for sciatic nerves range from 500 kPa to 8.1 MPa.^41,42^ In this context, P­(3HO) represents a promising member of the PHAs group for the fabrication of NGCs. Other PHAs, such as P­(3HB), as well as P­(3HB)/P­(3HO) blends, exhibit substantially higher *E*.^7,17^ For BTE, softer substrates have also demonstrated biological advantages. Zhang et al. demonstrated, in *in vitro* studies using MC3T3-E1 cells, that osteogenic differentiation was enhanced on matrices with elastic moduli ranging between 0.6 and 2.7 MPa.^43^ Nevertheless, materials used for the reconstruction of critical bone defects, particularly metallic implants, typically possess *E* in the range of several hundred GPa.^36,44^ Qin et al. further demonstrated that tailoring the *E* of Ti6Al4V to approximately 13 MPa accelerates osteogenesis by improving mechanical adaptability during bone regeneration, while higher-modulus phases (∼hundreds of MPa) ensure sufficient load-bearing capacity.^36^ In this regard, P­(3HO) appears to be a highly promising coating material for metallic bone implants.

Another critical surface-related parameter of implantable materials is wettability and surface free energy, as these properties govern initial cellular responses at the implant interface by modulating protein adsorption, cell adhesion, differentiation, and proliferation, and by influencing immunomodulatory processes.^45^ Accordingly, surface wettability was evaluated using water (Figure 2f and Table S4) and diiodomethane (Figure S7 and Table S4). These measurements were subsequently used to calculate the polar (Figure S8 and Table S5) and dispersive (Figure S9 and Table S5) components, as well as the total surface tension (Figure 2g and Table S5). The water contact angle of pristine P­(3HO) was 79.5°, which is comparable to values reported in the literature for other representatives of PHAs, i.e., P­(3HB): 80.9°.^46^ Notably, statistically significant changes in water wettability were observed only for the P­(3HO)_Zn/Al-turm, for which the contact angle decreased by 17% relative to P­(3HO). Hydrophobicity is generally considered unfavorable for implant applications, as enhanced hydrophilicity promotes protein adsorption, triggering a cascade of biological events that ultimately govern tissue-implant integration.^45^ Interestingly, distinct trends were observed for surface tension. For the turm-functionalized nanocomposites, P­(3HO)_Zn/Al-turm and P­(3HO)_Ca/Al-turm, a reduction in surface free energy of 24% and 22%, respectively, was recorded (Figure 2h and Table S5). This effect can be attributed to the chemical structure of turm, which contains hydroxyl, aromatic, and carbonyl groups that interact with the P­(3HO) matrix, thereby lowering the surface tension.^47^ In contrast, the nonfunctionalized nanocomposites, P­(3HO)_ZnO NPs and P­(3HO)_Ca/Al, exhibited increases in surface free energy of 21% and 8%, respectively.

### 3.3 Thermal Properties Relevant to Additive Manufacturing

Thermal properties are key parameters in evaluating newly developed polymer compositions, particularly the glass transition temperature (*T*_*g*_), melting temperature (*T*_*m*_), and onset of thermal degradation (*T*_*onset*_). These parameters are critical for materials intended for future use in additive manufacturing technologies such as FDM or FFF.^48,49^ Specifically, *T*_*g*_ defines the transition from a glassy (rigid) to a rubbery state, directly influencing melt flow behavior and interlayer adhesion during printing. Furthermore, *T*_*m*_ determines the processing window required to achieve sufficient plasticization without inducing thermal degradation, while *T*_*onset*_ defines the upper temperature limit for safe processing. Together, these parameters enable rational selection of processing conditions that ensure stable material extrusion while preserving polymer integrity.^9,48^ The incorporation of functionalized turmeric-based nanoparticles resulted in lack of changes in *T*_*g*_ for the P­(3HO)_ZnO NPs, whereas a slight reduction in *T*_*g*_ was observed for all other samples compared to neat P­(3HO): P­(3HO)_Zn/Al-turm, P­(3HO)_Ca/Al and P­(3HO)_Ca/Al-turm (Table 1 and Figures S14–S16). Furthermore, two *T*_*g*_ were observed for P­(3HO)_Zn/Al-turm: −50.9 and −40.3 °C. Similar observations were made in the study by Chen et al. In this case, the occurrence of two glass transition temperatures in the poly­(vinyl alcohol) (PVA)/silica (SiO_2_) nanocomposite reflects the coexistence of mobile PVA chains in the bulk matrix and constrained chains in an interfacial layer strongly interacting with the SiO_2_ surface.^50^ Moreover, *T*_*m*_ and *T*_*onset*_ remained largely unaffected across all investigated compositions, indicating that nanoparticle incorporation does not compromise the thermal stability of the polymer matrix (Table 1 and Figures S10–S16). Importantly, all composites exhibited an increase in the degree of crystallinity (*X*_*c*_), reaching approximately 4% for both P­(3HO)_Ca/Al and P­(3HO)_Ca/Al-turm systems. The increase in crystallinity, particularly evident in the case of P­(3HO)-_Ca/Al-turm, may be directly related to the observed decrease in *E* (Table 1 and Figure 2f), which may be caused by the uneven dispersion of the filler in the polymer matrix. Similar structure–property relationships were reported by Lizarraga-Valderrama et al., who demonstrated that the addition of bioactive glass (BG45S5) to PHAs resulted in changes in crystallinity accompanied by a 2–5-fold decrease in elastic modulus. Notably, the effect on crystallinity was governed by the chemical composition of the filler rather than its concentration (0.5–2.5 wt.%).^7^ From a processing perspective, the minor variations observed in *T*_*g*_, *T*_*m*_, and *T*_*onset*_ do not necessitate modification of standard FDM or FFF processing temperatures. Provided that appropriate melt rheological conditions are maintained within the operational window, the materials are expected to exhibit stable extrusion behavior. This thermal robustness is particularly advantageous for the future application of the developed compositions in the additive manufacturing of functional biomedical structures.^51^

### 3.4 Composition-Dependent Indirect Cytotoxicity

Indirect cytotoxicity assays were performed to evaluate whether compounds leaching from the fabricated nanocomposites exert toxic effects on cells. Potentially cytotoxic species include residual organic solvents from the synthesis process, intermediate reaction products, material degradation products, as well as nanoparticles and ions released during 24 h incubation in cell culture medium.^9^ Materials intended for BTE and NTE were evaluated using MC3T3-E1 and NG108-15 cell lines cultured in α-MEM and DMEM, respectively. All experiments were conducted in accordance with ISO 10993-5, with extracts prepared following ISO 10993-12 guidelines. After 24 h exposure of MC3T3-E1 and NG108-15 cells to the obtained extracts, cellular metabolic activity was quantified using a resazurin-based assay. Cell viability values were normalized to a negative control consisting of cells cultured on polystyrene (PS).

Quantitative analysis (Figure 3a and Table S6) revealed that MC3T3-E1 cell viability remained comparable to or slightly higher than the negative control for P­(3HO), P­(3HO)_ZnO NPs, and P­(3HO)_Ca/Al. In contrast, exposure to extracts derived from P­(3HO)_Zn/Al-turm and P­(3HO)_Ca/Al-turm nanocomposites resulted in a moderate reduction in cell viability, by approximately 21% and 14%, respectively. Importantly, these values remain well above the cytotoxicity threshold defined by ISO 10993-5, which considers a reduction in viability exceeding 30% as indicative of cytotoxic effects, confirming the overall cytocompatibility of the materials.^9^ For the NG108-15 neuronal cell line, cell viability was comparable to the negative control across all investigated materials (Figure 4a and Table S6). The maintained or slightly enhanced viability observed for selected samples can be attributed to multiple factors. First, (*R*)-3-hydroxycarboxylic acids released during the degradation of P­(3HO) have been reported to stimulate cellular viability.^13^ Moreover, Luo et al. demonstrated that divalent metal cations can promote bone formation by modulating sensory and sympathetic nervous system activity. Consistent with this, cytotoxicity studies indicate that relatively high concentrations of Zn^2+^ and Ca^2+^ ions do not induce cell death, with reported threshold concentrations of 11.8 μM and 20 μM, respectively.^52^ Notably, elevated Ca^2+^ concentrations are closely associated with pro-regenerative effects in neural tissue, as Ca^2+^ ions regulate key stages of axonal regeneration, including postinjury membrane resealing, growth cone formation and stabilization, and local protein synthesis within the axon.^53^ Similarly, Li et al. reported that Zn^2+^ ion release plays a dominant role in enhancing bone regeneration by stimulating bone marrow mesenchymal stem cell (BMSC) proliferation, increasing alkaline phosphatase (ALP) activity, and promoting mineralization, as evidenced by calcium deposition.^54^ The biological performance of the materials is further supported by the well-documented bioactivity of cur. Dahiya et al. demonstrated that cur does not induce cytotoxic effects in human fetal osteoblast (hFOB 1.19) cultures, as confirmed by live/dead assays, increased cell viability, enhanced ALP activity, and mineralization. These findings suggest that cur-containing composites provide a cell-compatible and osteogenic microenvironment.^2^ Moreover, Sun et al. reported that cur-loaded composite hydrogels exhibit no significant cytotoxicity toward RSC96 Schwann cells, with cell viability remaining above 70%, in accordance with ISO 10993-5, confirming the suitability of cur incorporation for applications in neural regeneration.^25^

**Figure 3:**
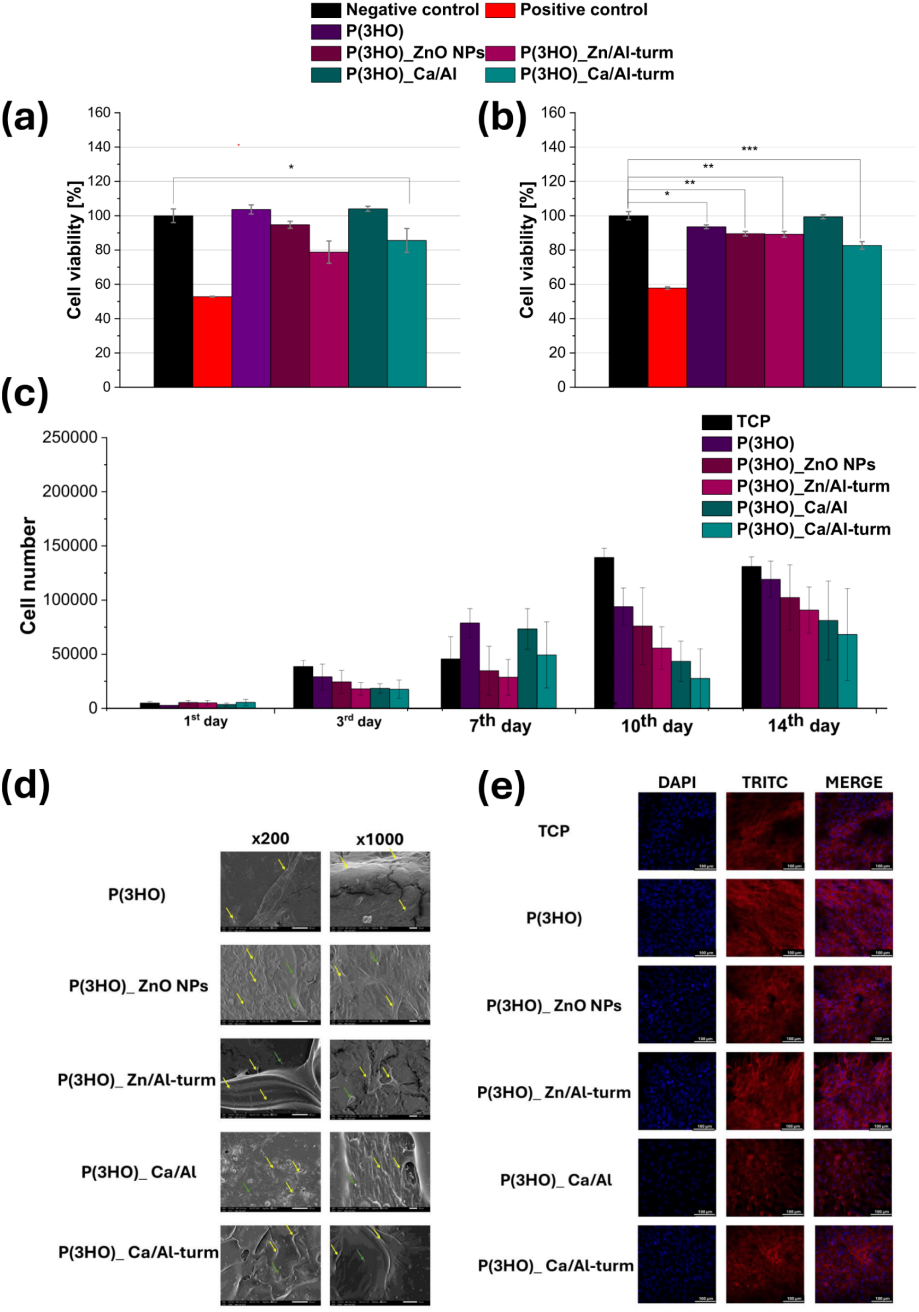
**(a)** Indirect cytotoxicity assessment of MC3T3-E1 cells exposed to material extracts obtained after 24 h incubation of the samples (concentration: 100 mg mL^–1^) (*n* ≥ 5). **(b)** Direct cytotoxicity results for MC3T3-E1 cells cultured in direct contact with the biomaterial surfaces for 2 days (*n* ≥ 5). Data are presented as mean ± SE. **(c)** Direct proliferation assay showing the temporal evolution of MC3T3-E1 cells number during 14 days of culture on the biomaterial surfaces, evaluated on days 1, 3, 7, 10, and 14 (*n* ≥ 3). Data are presented as mean ± SD. **(d)** Morphological analysis of MC3T3-E1 cells adhered to the nanocomposite surfaces after 14 days of culture; yellow arrows indicate MC3T3-E1 cells. **(e)** Fluorescence microscopy images of MC3T3-E1 cells after 14 days of direct proliferation assays, showing cell nuclei stained with DAPI and the cytoskeleton visualized with phalloidin-TRITC. Statistical significance is indicated as *p < 0.05, **p < 0.01, and ***p < 0.001.

**Figure 4:**
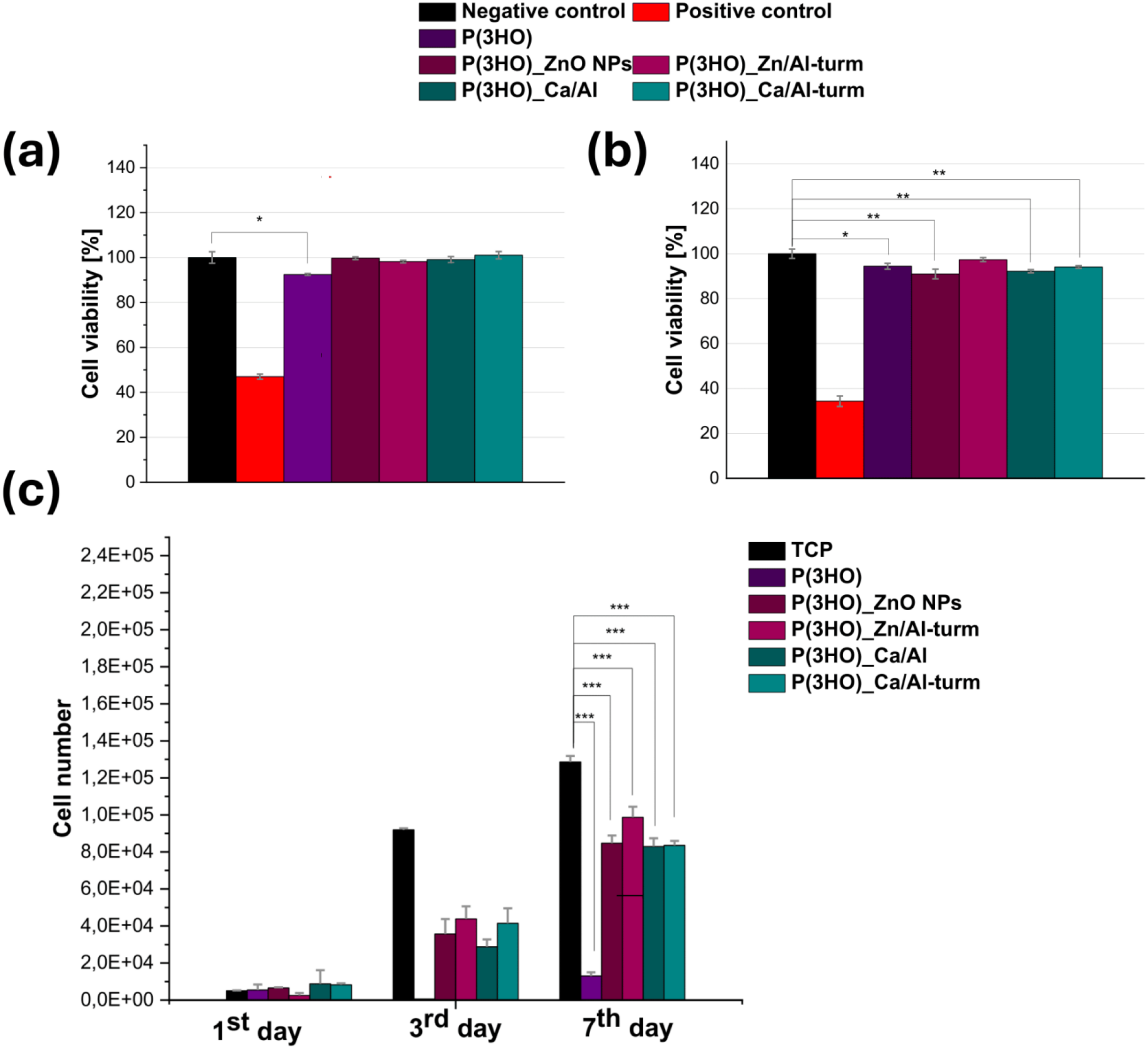
**(a)** Evaluation of indirect cytotoxicity toward NG108-15 cells following exposure to extracts prepared by incubating the materials for 24 h (100 mg mL^–1^) (*n* ≥ 5). **(b)** Direct cytotoxicity of NG108-15 cells cultured in direct contact with the biomaterial surfaces for 2 days (*n* ≥ 5). **(c)** Direct cell proliferation analysis illustrating changes in NG108-15 cell numbers over a 7-day culture period on the biomaterial surfaces, assessed on the 1^st^, 3^rd^, and 7^th^ day (*n* ≥ 3). Notably, after 2 days of culture, the medium was replaced with Fetal Bovine Serum (FBS)-free DMEM to induce neuronal differentiation of NG108-15 cells.^6,7,17,57^ Data are presented as mean ± SE. Statistical significance is denoted as *p < 0.05, **p < 0.01, and ***p < 0.001.

### 3.5 Adhesion, and Proliferation of MC3T-E1 and NG108-15 Cells on P­(3HO)-Based Nanocomposites

Direct cytotoxicity assays based on resazurin reduction, in which cells were seeded directly onto the material surfaces, were also conducted for both MC3T3-E1 (Figure 3b) and NG108-15 cells (Figure 4b). For all investigated materials, no cytotoxic effects were observed, as cell viability remained above 70%, in accordance with ISO 10993-5. For MC3T3-E1 cells, viability values were slightly lower than those of the negative control (Table S7), with statistically significant differences observed for P­(3HO), P­(3HO)_ZnO NPs, P­(3HO)_Zn/Al-turm, and P­(3HO)_Ca/Al-turm. A similar trend was observed for NG108-15 cells, where viability was modestly reduced compared to the negative control (Table S7). Statistically significant differences were detected for P­(3HO), P­(3HO)_ZnO NPs, P­(3HO)_Ca/Al, and P­(3HO)_Ca/Al-turm. Importantly, despite these differences, cell viability for all materials remained well above the cytotoxicity threshold, confirming cytocompatibility under direct-contact conditions. As reported in our previous work, outcomes of direct cytotoxicity assays are closely linked to cell adhesion to material surfaces.^8^ Accordingly, the minor reductions in viability observed here likely reflect active cell-material interactions rather than adverse cytotoxic effects. Overall, the surface structure and physicochemical properties of all investigated materials support cell adhesion of both MC3T3-E1 and NG108-15 cell lines, which is essential for regenerative applications.

In the next step, quantitative direct proliferation assays based on resazurin reduction were performed, in which cells were seeded directly onto the scaffold surfaces, and their metabolic activity was monitored at defined time points. For MC3T3-E1 cells, measurements were carried out on 1^st^, 3^rd^, 7^th^, 10_^th^_, and 14^th^ days, whereas for NG108-15 cells, they were performed on first, fourth, and seventh days.

The measured metabolic activity was subsequently converted into the corresponding number of cells present on the material surfaces. The direct proliferation results for MC3T3-E1 cells were fully consistent with the outcomes of the direct cytotoxicity assays. For all investigated materials, a time-dependent increase in cell number was observed, although the proliferation rate differed among the substrates. After 14 days of culture, the highest number of cells was detected on nanocomposites based on pristine P­(3HO), followed by scaffolds containing Zn^2+^ ions (P­(3HO)_ZnO NPs and P­(3HO)_Zn/Al-turm), whereas the lowest cell numbers were observed on Ca^2+^-containing nanocomposite films (P­(3HO)_Ca/Al and P­(3HO)_Ca/Al-turm) (Figure 3c). Notably, these quantitative differences were not reflected in microscopic (qualitative) observations. Both SEM imaging (Figure 3d) and confocal microscopy following staining of the actin cytoskeleton (Phalloidin-TRITC) and cell nuclei (DAPI) (Figure 3e) revealed comparable cell coverage, morphology, and cytoskeletal organization across all substrates. What is important, nanocomposites incorporating Zn/Al and Ca/Al species are well recognized for their potential to support bone tissue regeneration. Kang et al. demonstrated that Zn/Al stimulates the expression of osteogenesis-related genes *via* activation of the MAPK signaling pathway during proliferation, leading to enhanced synthesis of bone matrix proteins, including collagen, and increased mineralization by MC3T3-E1 cells.^4^ In a subsequent study, Fayyazbakhsh et al. confirmed the mineralization-inducing capacity, osteogenic activity, and biocompatibility of Ca/Al-containing materials.^55^ Consistently, Dahiya et al. reported that tricalcium phosphate (TCP) scaffolds containing cur significantly enhance the proliferation of hFOB 1.19 cells compared to pristine TCP-based materials.^2,56^

Direct proliferation assays of NG108-15 cells (Figure 4c) were not fully consistent with the direct cytotoxicity results (Figure 4b) for the neat P­(3HO) film. Although NG108-15 cells readily adhered to the surface of P­(3HO), neither neuronal differentiation nor cell proliferation was observed. This behavior is in agreement with previous reports showing that P­(3HO) films fabricated by the SC technique support significantly fewer neurites compared to P­(3HO)/P­(3HB) blend films prepared at 50:50 and 25:75 ratios.^57^ Notably, P­(3HO)-based films can be rendered permissive for neuronal differentiation and proliferation by incorporating suitable additives. As shown in Figure 4c, all nanocomposites (P­(3HO)_ZnO NPs, P­(3HO)_Zn/Al-turm, P­(3HO)_Ca/Al, and P­(3HO)_Ca/Al-turm) exhibited a time-dependent increase in neuronal cell number. Among them, P­(3HO)_Zn/Al-turm showed the highest proliferation rate, which is likely attributable to a synergistic effect between Zn/Al-LDH and turm.

This interpretation is supported by previous studies. Sun et al. demonstrated that local delivery of cur from a keratin-chitosan hydrogel exerts a direct regenerative effect on nerve tissue while preserving excellent biocompatibility and material stability, as confirmed by *in vivo* behavioral, electrophysiological, and histological analyses.^25^ Similarly, Delavar et al. reported that sustained release of cur from a piezoelectric fibrous nerve conduit significantly enhanced sciatic nerve regeneration, increasing axon number and promoting perineurium reconstruction in a rat model.^3^

### 3.6 Composition-Driven Immunomodulatory Macrophages Response

Macrophages are central regulators of the immune response, exhibiting pronounced plasticity that enables dynamic adaptation to microenvironmental cues. Depending on local stimuli, macrophages predominantly polarize toward a pro-inflammatory (M1) or an anti-inflammatory and pro-regenerative (M2) phenotype. While M1 macrophages support host defense through the secretion of pro-inflammatory cytokines, M2 macrophages promote inflammation resolution, angiogenesis, and bone remodeling. Importantly, macrophage polarization directly influences osteoblast, osteoclast, and mesenchymal stem cell activity, thereby playing a pivotal role in bone homeostasis.^58^ In PNI, macrophage recruitment is a key event in both regenerative processes and the development of neuropathic pain, with macrophages infiltrating the lesion site within days following injury in response to Schwann cell-derived signals.^59^ The immunomodulatory effects of the developed nanocomposites were evaluated using human monocyte-derived macrophages (THP-1, Figure 5a). Following PMA-induced differentiation into nonpolarized macrophages (M0), cells were cultured directly on the nanocomposite surfaces, and their secretory profiles were compared with those of conventionally induced M1 and M2 macrophage controls. Transforming growth factor β1 (TGF-β1) and matrix metalloproteinase-2 (MMP-2) were selected as key markers due to their established roles in inflammation resolution, tissue remodeling, osteogenic differentiation, Schwann cell modulation, and axonal regeneration.^59,60^

**Figure 5:**
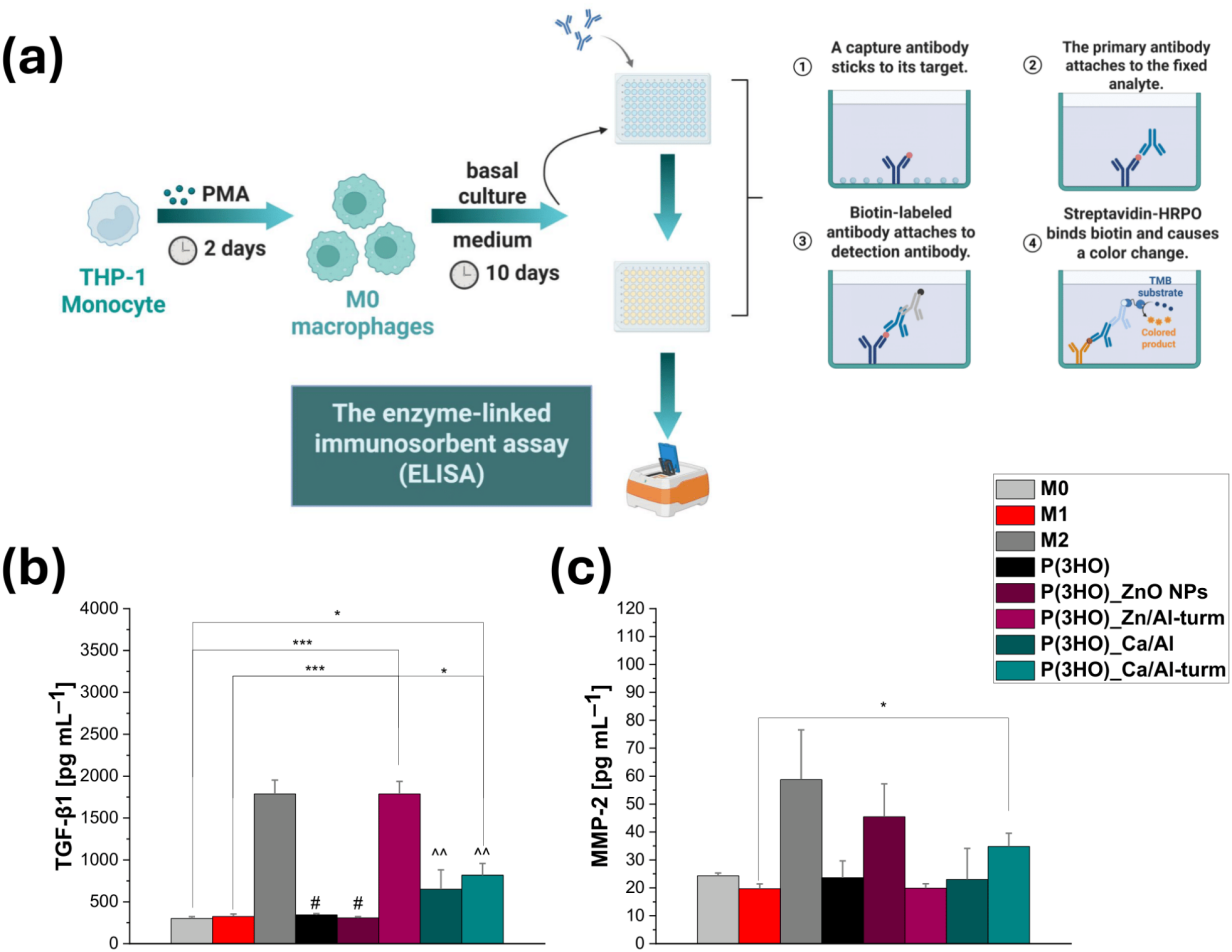
**(a)** Schematic illustration of the experimental workflow used to evaluate the anti-inflammatory potential of the prepared materials through the assessment of TGF-β1 and MMP-2 secretion. Human THP-1 monocytes were differentiated into adherent macrophages by treatment with phorbol 12-myristate 13-acetate (PMA) for 2 days, yielding nonpolarized macrophages (M0). Subsequently, the cells were cultured directly on the material surfaces for 10 days. In parallel, control cultures were prepared: untreated macrophages maintained for 10 days (M0 control); pro-inflammatory macrophages (M1), generated by stimulation with lipopolysaccharide (LPS) and interferon-γ (IFN-γ) for 3 days followed by 7 days of culture in fresh medium; and anti-inflammatory macrophages (M2), obtained by stimulation with interleukin-4 (IL-4) and interleukin-13 (IL-13) for 3 days, followed by 7 days of culture in fresh medium. During the 7-day continuation phase, half of the culture medium was replaced with fresh RPMI-1640 every 2 days. After completion of the culture period, supernatants were collected, and the concentrations of TGF-β1 and MMP-2 were quantified using enzyme-linked immunosorbent assay (ELISA). The ELISA procedure involved: (1) binding of the capture antibody to the target analyte, (2) attachment of the primary antibody to the immobilized analyte, (3) binding of a biotin-labeled detection antibody, and (4) streptavidin–HRP-mediated signal generation resulting in a colorimetric response. Created with BioRender.com **(b)** Quantification of TGF-β1 (*n* = 3) and **(c)** MMP-2 levels (*n* = 3) determined in the conducted experiments. Data are presented as mean ± SE. Statistical significance between groups is indicated as *p < 0.05, **p < 0.01, and ***p < 0.001.

Macrophages cultured on the P­(3HO)_Zn/Al-turm nanocomposite secreted TGF-β1 at levels comparable to the anti-inflammatory M2 control, indicating a pronounced shift toward an immunoregulatory phenotype. For MMP-2, the highest secretion induced by nanocomposite exposure remained approximately 24% lower than that of the M2 control, suggesting controlled matrix remodeling. Similarly, exposure to the P­(3HO)_Ca/Al-turm nanocomposite resulted in statistically significant increases in both TGF-β1 (818.0 ± 140.6 pg mL^–1^) and MMP-2 (34.8 ± 4.8 pg mL^–1^) secretion compared to the pro-inflammatory M1 control, corresponding to increases of 152% and 77%, respectively, while remaining substantially lower than M2 levels (Figure 5b,c and Table S8).

Matrix metalloproteinases, including MMP-2, play a dual role in regeneration by enabling extracellular matrix remodeling and regulating growth factor bioavailability, including TGF-β1. However, excessive MMP-2 activity may exacerbate inflammatory damage, highlighting the importance of balanced secretion. The moderate elevation of MMP-2 observed for turm-modified nanocomposites, therefore, suggests a favorable immunoregulatory profile rather than a pro-inflammatory response.^61^

Collectively, these findings demonstrate that nanocomposites incorporating turm-modified nanoparticles (Zn/Al-turm and Ca/Al-turm) promote a pro-regenerative macrophage response characterized by elevated TGF-β1 and moderately increased MMP-2 secretion, approaching, but not exceeding, the M2 phenotype. This behavior is consistent with the absence of cytotoxic effects observed in indirect cytotoxicity assays and aligns with previous *in vivo* observations, where P­(3HO)-based wound dressings significantly enhanced angiogenesis despite reduced TGF-β1 mRNA expression.^10^ Consistent with these observations, Rashid et al. developed composite films based on poly­(vinyl alcohol), gelatin, and tannic acid, supplemented with cur. They demonstrated their anti-inflammatory activity during wound healing. The cur-containing dressings markedly reduced the expression of pro-inflammatory cytokines, with TNF-α and IL-6 levels decreased by 76% and 68%, respectively, compared to untreated controls.^62^ Similarly, Wang et al. reported that composite dressings fabricated from glycyrrhizic acid-derived polymers, cur, and silver nanoparticles effectively promoted macrophage polarization toward the M2 phenotype and suppressed pro-inflammatory cytokine expression.^63^ Taken together, the above results underscore the potential of cur-functionalized P­(3HO)-based nanocomposites to actively modulate the immune microenvironment, thereby supporting coordinated bone and nerve tissue regeneration.

## 4 Overall Discussion

The physicochemical, structural, mechanical and surface properties of materials intended for tissue regeneration, particularly coatings used on implants, determine their ability to initiate and modulate the biological response. This mainly includes the ability to promote cell adhesion, differentiation and proliferation, as well as the regulation of inflammatory processes.^9,33,35,36,45^ Commercially available polymeric nerve conduits, such as NeuraGen, NeuroMatrix, Neurotube, Silastic and Neurolac, represent a particularly interesting group. According to a review by Chan et al., these materials still do not simultaneously provide optimal bioactivity, mechanical properties and control of the inflammatory response.^64^ In recent years, extensive research has also been conducted into the possibility of replacing hydroxyapatite materials used in coatings for bone tissue regeneration.^65^ At the same time, a common side effect of both bone and nerve implantation is the induction of an immunological response, often caused by the release of polymer degradation products.^8^ In this study, PHA polymers were used to produce coatings for bone tissue implants and nerve conduits. These polymers stand out from other biodegradable materials due to their ability to degrade under both aerobic and anaerobic conditions, which is particularly important in the complex environment of the human body. In biological systems, PHA degradation occurs mainly *via* enzymatic and nonenzymatic hydrolysis. Based on Cichoń et al., we propose that, in the case of the developed implants, the degradation process will proceed*via* gradual surface erosion, which will enable controlled decomposition of the material and the release of turm.^11^ Another important aspect is that, as the polymer coating degrades, molecules of 3-(*R*)-hydroxycarboxylic acids are released; these are natural metabolites produced by the body *via* the β-oxidation pathway, which confirms their high biocompatibility and lack of toxicity.^8–11,13^ This was further confirmed by our direct cytotoxicity assays on the MC3T3-E1 (Figure 3a and Table S6) and NG108-15 (Figure 4a and Table S6) cell lines. Importantly, this study demonstrates that by utilizing P3HO and LDHs-based nanomaterials, it is possible to design materials in accordance with the concept of nanoarchitectonics. This approach involves not only the control of macroscopic properties, but also the multilevel organization of the structure, encompassing the presence of nanoparticles, their agglomerates (Figure 2a–d) and the resulting heterogeneous surface topography (Figure 2e and Table 1), which influences protein adsorption and cell adhesion.^66^ This approach enabled the design of a surface conducive to cell adhesion, both for MC3T3-E1 (Figure 3b and Table S7) and, particularly importantly, for NG108-15 (Figure 4b and Table S7). The introduction of nanofillers enabled the creation of structures that promote the differentiation and proliferation of NG108-15 cells into neurons, which was not possible with films made exclusively from the native polymer (Figures 4c and S17). The excellent biocompatibility demonstrated in this study was also previously confirmed in *in vivo* studies conducted on a mouse model, in the context of research into wound dressings.^10^ From the perspective of materials chemistry, the presence of ions such as Ca^2+^ and Zn^2+^, as well as turm, released during the gradual degradation of materials, may enable the modulation of cellular processes, including proliferation and differentiation.^2,25,52–54^ However, the key aspect is integrating all these factors to achieve a controlled immune response. The nanocomposites developed promote the polarization of macrophages toward the M2 phenotype, indicating their ability to actively shape a microenvironment conducive to regeneration. In summary, the nanocomposites presented in this study, developed in accordance with the concept of nanoarchitectonics, open new prospects for the design of PHA-based materials, i.e. P­(3HO), particularly for their application in peripheral nerve regeneration.

## 5 Future Perspectives

The results of this study demonstrate that the developed polymer nanocomposites P­(3HO)_Zn/Al-turm and P­(3HO)_Ca/Al-turm possess significant biomedical potential, particularly for applications in BTE and NTE. Future work should focus on enabling their practical implementation in additive manufacturing technologies such as fused deposition modeling (FDM) or fused filament fabrication (FFF), which will require homogeneous polymer-filler dispersion achieved *via* counter-rotating twin-screw extrusion, followed by filament fabrication using a dedicated compounding process. Additive manufacturing technologies, such as FDM and FFF, are redefining the fabrication of patient-specific platforms for regenerative medicine by enabling unprecedented control over geometry and structural hierarchy. However, the integration of biofunctional polymer-based nanocomposites into filament-based additive manufacturing remains largely unexplored, primarily due to the limited availability of materials that simultaneously meet processing and biological requirements.^67,68^

In particular, the nanocomposites could be employed as printed surface layers or coating systems for load-bearing implants manufactured from metallic alloys. While such implants provide the mechanical strength required for load transfer, they often exhibit suboptimal biomechanical compatibility and limited biointeractivity. Among metallic biomaterials, magnesium-based alloys are of particular interest due to their elastic modulus (41–45 GPa), which closely matches that of natural bone, making them attractive candidates for orthopedic implant applications. However, their clinical translation remains limited by rapid corrosion, leading to premature loss of mechanical integrity, excessive hydrogen evolution, local alkalization, and an increased risk of early inflammatory responses following implantation.^69,70^ The application of nanocomposite coatings developed in this work could effectively address these limitations. Incubation studies demonstrated that the nanocomposite films retained their structural integrity without morphological degradation when exposed to SBF and Ringer’s solution, highlighting their stability under physiologically relevant conditions. Moreover, the nanocomposites offer the possibility of incorporating bioactive agents that can modulate the host immune response. In particular, the P­(3HO)_Zn/Al-turm nanocomposite promoted macrophage polarization toward the pro-regenerative M2 phenotype, suggesting its potential to enhance tissue healing and integration. Taken together, these findings open new avenues for the development of multifunctional, additively manufactured coating systems that combine mechanical compatibility, corrosion mitigation, and immunomodulatory activity, thereby advancing the next generation of bioactive implants for BTE and NTE.

## 6 Conclusions

The present study demonstrates that bacterially derived P­(3HO) can be transformed into a multifunctional and bioactive platform for BTE and NTE through rational nanocomposite design. All developed materials, including supported the adhesion of both preosteoblastic MC3T3-E1 and neuronal NG108-15 cells, with no cytotoxic effects observed under indirect-contact conditions. Importantly, all nanocomposites promoted robust proliferation of MC3T3-E1 cells, confirming their suitability for BTE, while the incorporation of inorganic fillers enabled P­(3HO) to overcome its intrinsic limitations toward neuronal growth and differentiation. Crucially, the introduction of both unmodified and turn-functionalized fillers fundamentally altered the biological performance of P­(3HO), converting a polymer that is otherwise poorly permissive for neuronal proliferation into a material that actively supports neuronal cell growth. In particular, P­(3HO)_Zn/Al-turm showed the most pronounced enhancement of NG108-15 proliferation, highlighting the ability to direct regenerative processes through compositional tuning. This demonstrates that precise control over the chemical composition and surface functionality of P­(3HO)-based nanocomposites enables targeted modulation of cell-material interactions, enabling the design of materials tailored to regenerative applications. Moreover, the turm-functionalized nanocomposites exhibited a distinct immunomodulatory profile. Both P­(3HO)_Zn/Al-turm and P­(3HO)_Ca/Al-turm induced a significant increase in TGF-β1 secretion by macrophages, accompanied by a moderate and controlled elevation of MMP-2, indicating a shift toward pro-regenerative, M2-like phenotype rather than a pro-inflammatory response. This balanced immunoregulatory activity is highly desirable for coordinated bone and nerve regeneration, while inflammation resolution, matrix remodeling, and growth factor signaling must be precisely arranged.

Taken together, these findings establish turm-functionalized LDH-based P­(3HO) nanocomposites as a new class of bioactive, immunomodulatory, and cytocompatible materials capable of simultaneously supporting osteogenic and neuroregenerative processes. The demonstrated ability to tune cell proliferation, adhesion, and immune response through nanocomposite engineering provides a powerful strategy for designing next-generation biomaterials for regenerative medicine and functional implant coatings.

## Supplementary Material



## Data Availability

Data for this article are available at the Mendeley Data repository at https://data.mendeley.com/datasets/nh2zwtcs8s/2 and https://data.mendeley.com/datasets/mtyv8pk35z/1.

## Notes

Raman point spectra of synthesized powders; textural parameters of the studied materials determined from nitrogen adsorption–desorption measurements; basal spacing (d_hkl_) of NPs; IR spectra and XRD diffractograms of nanocomposites; SEM micrographs of P­(3HO) and the nanocomposites; Young modulus (*E*) of the prepared nanocomposites; the wettability of the materials treated with diiodomethane and water; the polar and dispersive components of the surface tension; TGA and DSC analyses of nanocomposites; indirect and direct cytotoxicity of the prepared nanocomposites; surface morphology of the nanocomposites on day 7 of the proliferation assay (NG108-15 cell line); quantification of TGF-β1 and MMP-2 in Culture Supernatants of PMA-Differentiated THP-1 Macrophages after 10^th^ days of experiments (PDF)
