# Supplementary material for: Composite Nanoarchitectonics with Turmeric-Functionalized Layered Double Hydroxide/Poly(3-hydroxyoctanoate) as Bioactive Coatings for Bone and Nerve Regeneration
Source: ACS Appl Mater Interfaces. 2026 Apr 23;18(17):25287–305. doi: 10.1021/acsami.6c04793 (PMC13308884; doi:10.1021/acsami.6c04793)
Supplement: Supplementary file 1 [file am6c04793_si_001.pdf]

Supporting Information

Composite Nanoarchitectonics with Turmeric-Functionalised Layered Double Hydroxide/Poly(3-hydroxyoctanoate) as Bioactive Coatings for Bone and Nerve Regeneration

Katarzyna Haraźna[1], Sonia Bujok[2,3], Kamila Lis[4], Julita Wesołowska[5], Maciej Bik[6], Agnieszka M. Tomala[1], Karina Niziołek[1,4], Martina Nevoralová[3], Justyna Prajsnar[2], Hynek Beneš[3], Maciej Guzik[2], Agnieszka Sobczak-Kupiec[1]*

[1] Department of Materials Engineering, Faculty of Materials Engineering and Physics, 37 Jana Pawła II Av., Cracow University of Technology, 31-864 Cracow, Poland

[2] Jerzy Haber Institute of Catalysis and Surface Chemistry, Polish Academy of Sciences, Niezapominajek 8, 30-239 Cracow, Poland

[3] Institute of Macromolecular Chemistry, Czech Academy of Sciences, Heyrovského nám. 2, 162 00 Prague 6, Czech Republic

[4] CUT Doctoral School, Department of Materials Engineering, Faculty of Materials Engineering and Physics, 37 Jana Pawła II Av., Cracow University of Technology, 31-864 Cracow, Poland

[5] Laboratory of Microscopic Imaging, Maj Institute of Pharmacology, Polish Academy of Sciences, CEPHARES, 12 Smętna Street, 31-343 Cracow, Poland

[6] Faculty of Materials Science and Ceramics, AGH University of Krakow, 30-059 Cracow, Poland

Corresponding author: Katarzyna Haraźna, Department of Materials Engineering, Faculty of Materials Engineering and Physics, 37 Jana Pawła II Av., Cracow University of Technology, 31-864 Cracow, Poland

e-mail: katarzyna.harazna@pk.edu.pl

Table of the content

S1 Results S5

S1.1 Raman analysis of LDHs S5

Figure S1. Raman point spectra of synthesised powders S6

S1.2 BET analysis of obtained powders S7

Table S1. Textural parameters of the studied materials determined from nitrogen adsoption-desorption measurements S8

Table S2. Basal spacing (d_{hkl}) of NPs that exhibit layered structure: Zn/Al-turm, Ca/Al, and Ca/Al-turm. S9

Figure S2. IR spectrum of P(3HO), P(3HO)_Zn/Al-turm and Zn/Al-turm.. ....S10

Figure S3. IR spectrum of P(3HO), P(3HO)_Ca/Al-turm and Ca/Al-turm. S11

Figure S4. XRD of P(3HO), P(3HO)_Zn/Al-turm and Zn/Al-turm....S12

Figure S5. XRD of P(3HO), P(3HO)_Ca/Al-turm and Ca/Al-turm....S13

Figure S6. SEM micrographs of P(3HO) and the prepared active nanocomposites, P(3HO)_Zn/Al-turm and P(3HO)_Ca/Al-turm, before and after 41 days of incubation in Ringer's solution and simulated body fluid (SBF). S14

Table S3. Young modulus (E) of the prepared nanocomposites. S15

Figure S7. The wettability of the materials treated with diiodomethane. S16

Figure S8. The polar component of the surface tension. S17

Figure S9. The dispersive component of the surface tension S18

Table S4. The wettability of the nanocomposites treated with water and diiodomethane. S19

Table S5. The dispersive (γSP) and polar components (γSP), as well as the surface tension (γS) of the nanocomposites. S20

Figure S10. TGA curves of neat P(3HO) and P(3HO) nanocomposites containing Ca/Al (P(3HO)_Ca/Al), (C) Ca/Al-turm ((P(3HO)_Ca/Al-turm), and Ca/Al-turm. S21

Figure S11. DTG curves of neat P(3HO) and P(3HO) nanocomposites containing Ca/Al (P(3HO)_Ca/Al), (C) Ca/Al-turm ((P(3HO)_Ca/Al-turm), and Ca/Al-turm. S22

Figure S12. TGA curves of neat P(3HO) and P(3HO) nanocomposites containing ZnO NPs (P(3HO)_ZnO NPs), (C) Zn/Al-turm ((P(3HO)_Zn/Al-turm), and Zn/Al-turm. S23

Figure S13. DTG curves of neat P(3HO) and P(3HO) nanocomposites containing ZnO NPs (P(3HO)_ZnO NPs), (C) Zn/Al-turm ((P(3HO)_Zn/Al-turm), and Zn/Al-turm. S24

Figure S14. DSC curves (first heating) of P(3HO)-based nanocomposites. S25

Figure S15. DSC curves (cooling) of P(3HO)-based nanocomposites. S26

Figure S16. DSC curves (second heating) of P(3HO)-based nanocomposites. S27

Table S6. Indirect cytotoxicity of the prepared nanocomposites. S28

Table S7. Direct cytotoxicity of the prepared nanocomposites. S29

Figure S17. Surface morphology of the nanocomposites on day 7 of the proliferation assay. S30

Table S8. Quantification of TGF-β1 and MMP-2 in Culture Supernatants of PMA-Differentiated THP-1 Macrophages after 10th days of experiments. S31

Table S9. Composition of Simulated Body Fluid (SBF) and Ringer’s fluid. S32

S1 Results

S1.1 Raman analysis of LDHs

The structures of LDHs were fully confirmed by Raman spectroscopy (**Figure S1**). In the case of ZnO NPs, several Raman bands can be attributed to the nanoparticles, with the most characteristic E_2 modes observed at ca. 100 and 438 cm^{-1}, corresponding to Zn-related vibrations and the oxygen lattice vibrational mode, respectively[1,2]. Similarly, Ca/Al revealed three bands that can be attributed to this phase at around 534, 723 and 1057 cm^{-1} (the most characteristic) that come from the vibrations of Al–O–Ca linkages, NO_3^- ions and vibrations of anion intercalated in LDH material, respectively [3,4]. In case of turm-functionalized samples, a clearly noticeable influence of turmeric on the Raman spectra can be seen, as the raised background coming from the fluorescence effect can be observed. In order to reduce such effect, the laser bleaching was applied, and the resultant spectra revealed a number of characteristic bands. For Zn/Al-turm, one can observe several bands with the most relevant coming from turm at around 1600 and 1625 cm^{-1} attributed to stretching vibrations of C=C/C=O of an interring chain and to stretching C=C vibrations in aromatic ring, respectively. Another one at around 1130 cm^{-1} can be assigned to bending C–OH vibrations coupled to stretching (C=C-H) motions in the enolic group of the inter-ring chain. The last one at ca. 481 cm^{-1} comes from the skeletal CCC vibrations of the inter-ring chain and rings [5,6]. Furthermore, bands at around 554 and 1055 cm^{-1} come from LDH [3,4]. Very similar bands can be found for the Ca/Al-turm specimen with slightly less visible bands coming from the turm.

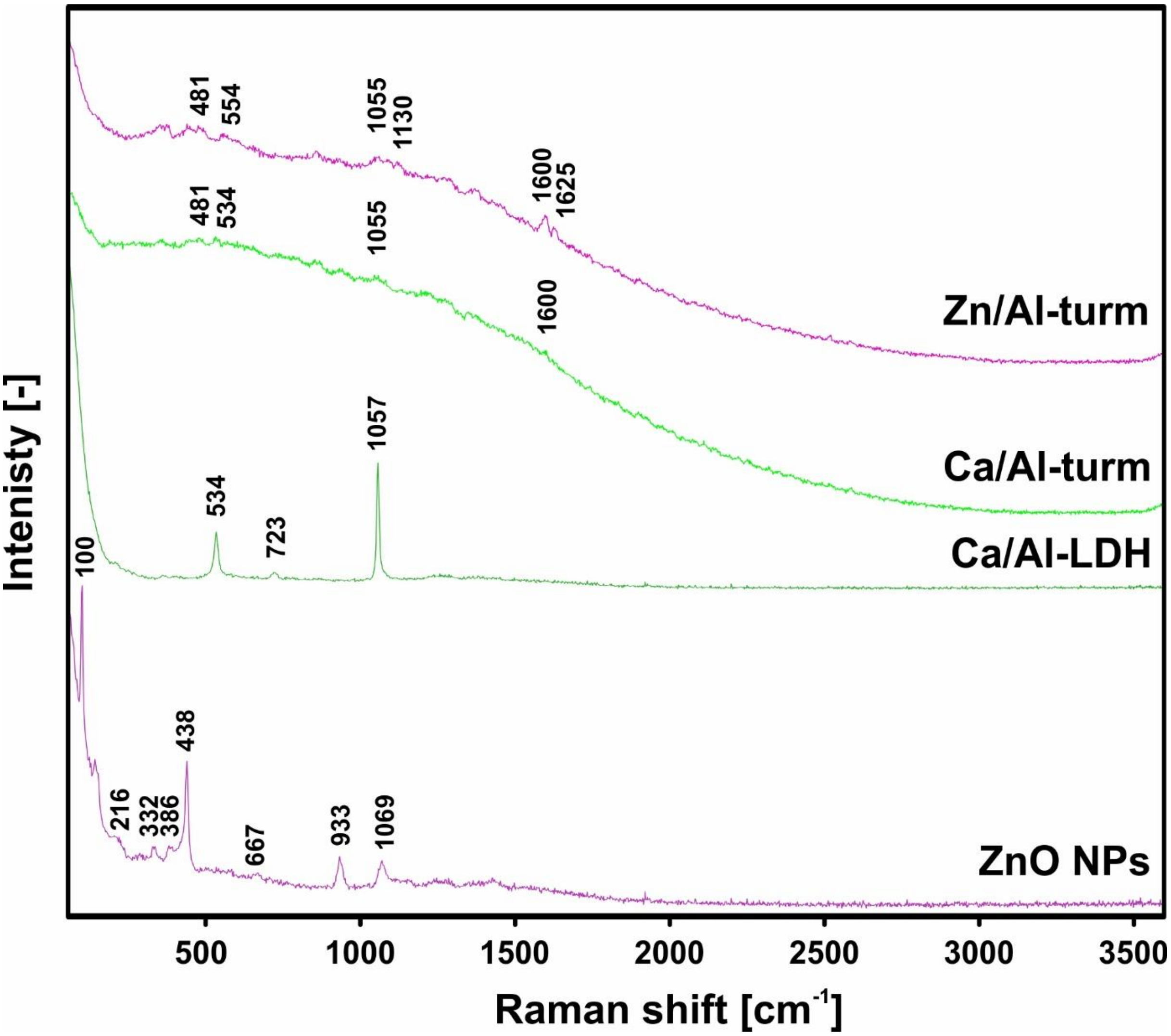

Figure S1. *Raman point spectra of synthesised LDHs*

S1.2 BET analysis of obtained powders

The specific surface area determined using the multipoint BET method for ZnO NPs, Ca/Al, Zn/Al–turm, and Ca/Al–turm was 8.4, 17.6, 14.0, and 13.6 m²/g, respectively. The t-plot analysis confirmed the complete absence of micropores in all samples (V_{mic} = 0 cm³/g). The external (nonmicroporous) surface area (S_{ext}) was identical to the BET surface area, indicating that the obtained nanoparticles are nonporous solids and that the measured surface area arises exclusively from the external geometry of the crystallites.

The pore size distribution calculated from the adsorption branch using the Barrett–Joyner–Halenda (BJH) method revealed dominant mesopore diameters of 15.7, 3.6, 8.9, and 3.6 nm for ZnO NPs, Ca/Al, Zn/Al–turm, and Ca/Al–turm, respectively. These results are in good agreement with those obtained using the Non-Local Density Functional Theory (NLDFT) model, which yielded modal pore sizes of 15.0, 3.8, 4.1, and 3.8 nm, respectively.

The application of the Frenkel–Halsey–Hill (FHH) model enabled the evaluation of surface roughness at the nanoscale. The calculated fractal dimension (D_s) was 2.4, 2.6, 2.5, and 2.6 for ZnO NPs, Ca/Al, Zn/Al–turm, and Ca/Al–turm, respectively, indicating a well-developed and irregular surface morphology of the nanoparticles.

In the case of Zn/Al–turm, a significant discrepancy between the dominant pore sizes determined by the BJH (8.9 nm) and NLDFT (4.1 nm) methods was observed. This difference arises from the thermodynamic limitations of the BJH model, which is based on the classical Kelvin equation and tends to overestimate mesopore sizes below 10 nm. In contrast, the NLDFT method, which accounts for the molecular density profile of the adsorbate near the adsorbent surface, is considered to provide a more accurate representation of the actual texture of the aggregates.

Table S1. *Textural parameters of the studied materials determined from nitrogen adsorption–desorption measurements.*

	**ZnO NPs**	**Ca/Al**	**Zn/Al-turm**	**Ca/Al-turm**
**S_{BET} (m^2/g)**	8.4	17.6	14.0	13.6
**S_{ext} (m^2/g)**	8.4	17.65	14.0	13.6
**V_{mic} (cm^3/g)**	0	0	0	0
**Pore diameter (nm)**	15.7	3.6	8.9	3.6
**Pore size distribution (nm)**	15.0	3.8	4.1	3.8
**Fitting Error (%)**	2.8	2.5	2.6	0.6
**D_s**	2.4	2.6	2.5	2.6

Where: S_{BET} - specific surface area determined using the multipoint BET method; S_{ext} - external surface area determined by the t-plot method (de Boer); V_{mic} - micropore volume determined by the t-plot method (de Boer); Pore diameter - average pore size determined using the Barrett–Joyner–Halenda method; Pore size distribution and fitting error - determined using Non-Local Density Functional Theory; Fractal dimension (D_s) - determined using the Frenkel–Halsey–Hill method.

Table S2. *Basal spacing (d_{hkl}) of NPs that exhibit layered structure: Zn/Al-turm, Ca/Al, and Ca/Al-turm* [7].

Sample	**2Θ [º]**	**(*hkl*)**	**d_{hkl} [nm]**
Zn/Al-turm	11.7	(003)	0.76
Ca/Al [7]	10.3	(002)	0.86
Ca/Al-turm	11.5	(002)	0.77

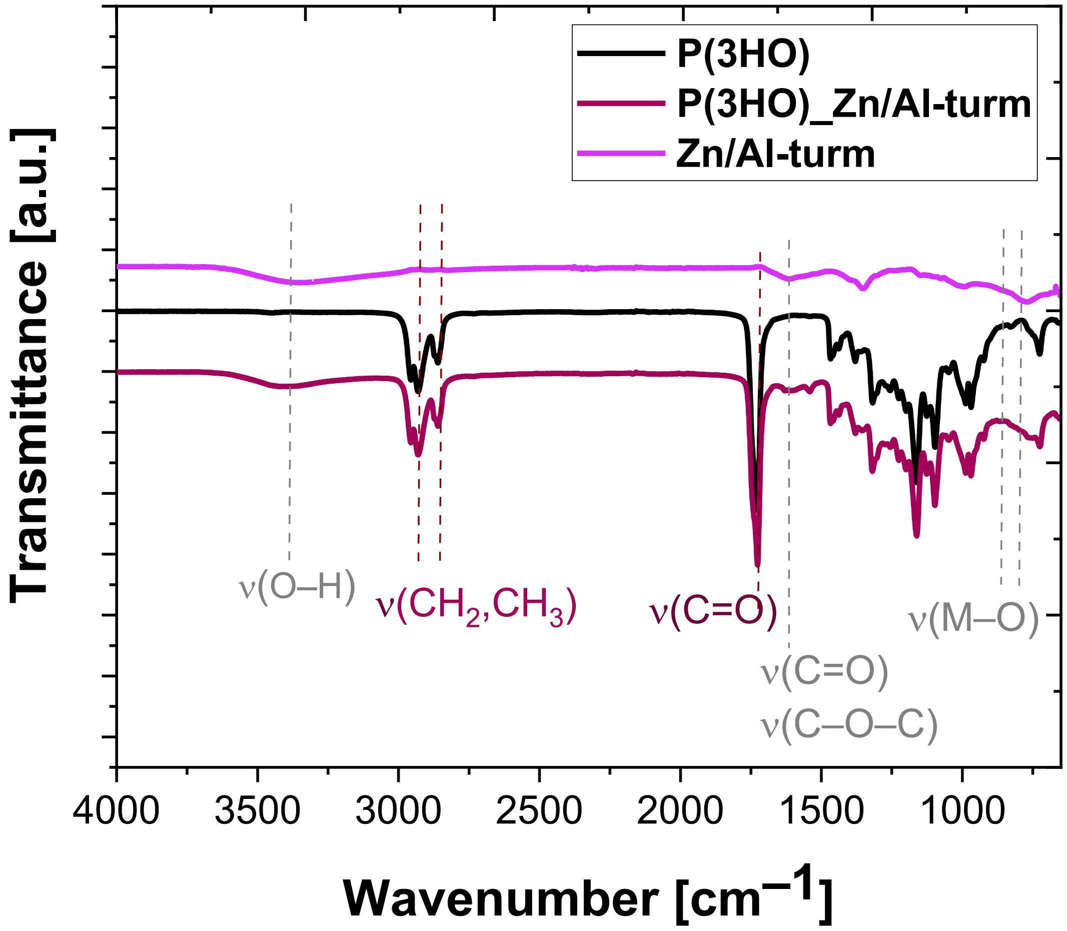

Figure S2. *IR spectrum of P(3HO), P(3HO)_Zn/Al-turm and Zn/Al-turm. Spectra of Zn/Al and P(3HO)_Zn/Al are presented in our previous work* [7].

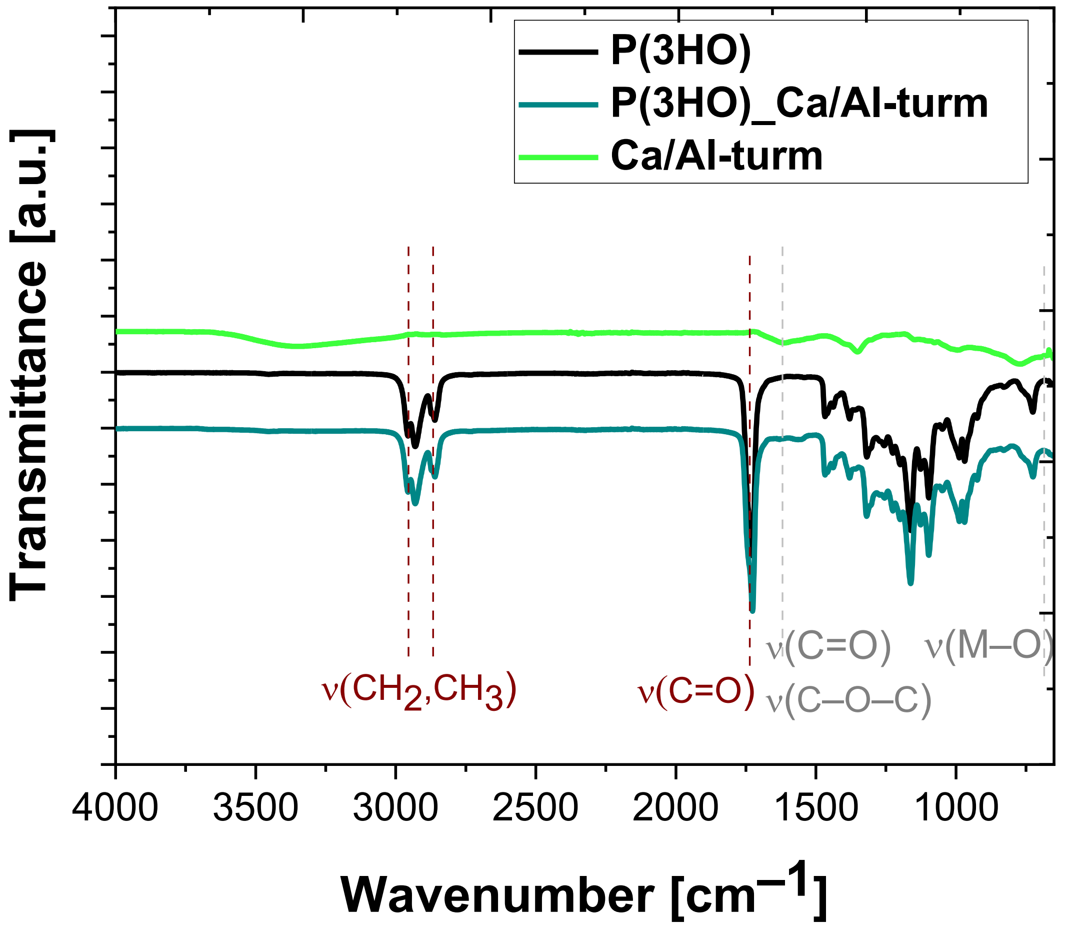

Figure S3. *IR spectrum of P(3HO), P(3HO)_Ca/Al-turm and Ca/Al-turm. Spectra of Ca/Al and P(3HO)_Ca/Al are presented in our previous work* [7].

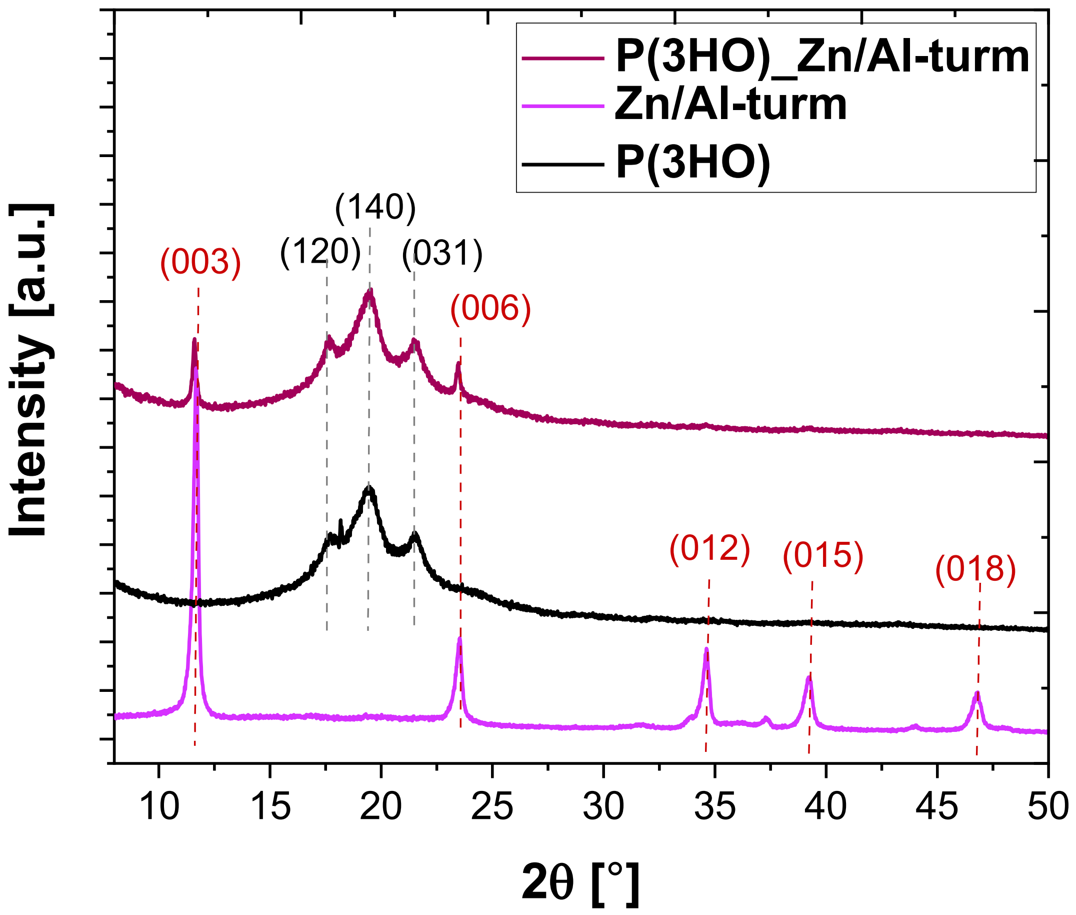

Figure S4. *XRD of P(3HO), P(3HO)_Zn/Al-turm and Zn/Al-turm. Diffractograms of Zn/Al and P(3HO)_Zn/Al are presented in our previous work* [7].

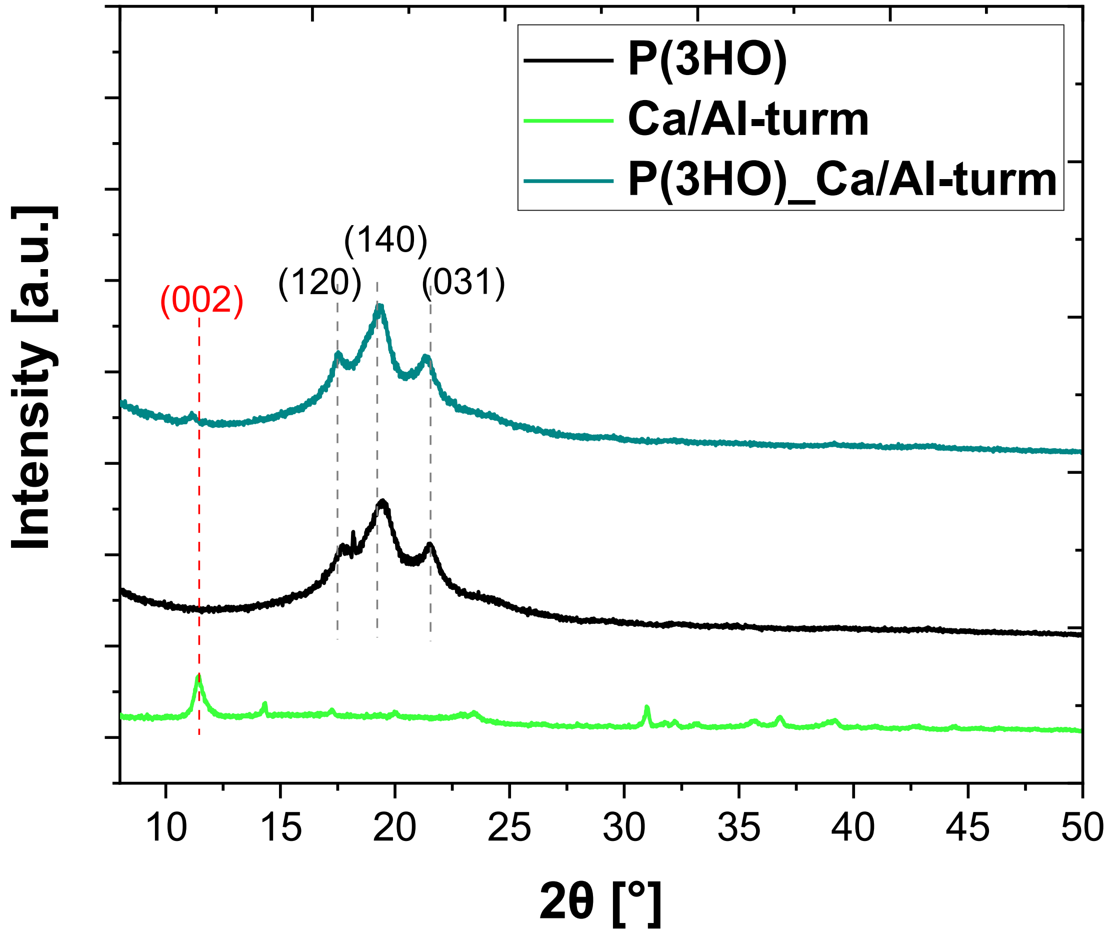

Figure S5. *XRD of P(3HO), P(3HO)_Ca/Al-turm and Ca/Al-turm. Diffractograms of Ca/Al and P(3HO)_Ca/Al are presented in our previous work* [7].

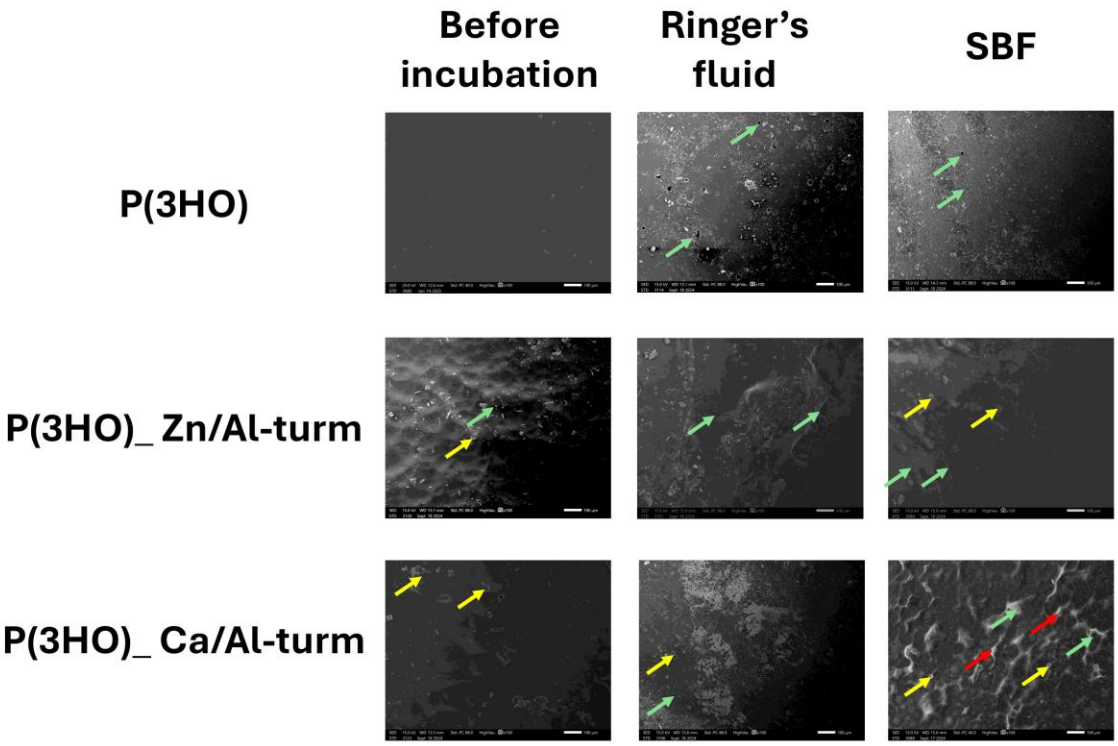

Figure S6. *SEM micrographs of P(3HO) and the prepared active nanocomposites, P(3HO)_Zn/Al-turm and P(3HO)_Ca/Al-turm, before and after 41 days of incubation in Ringer's solution and simulated body fluid (SBF). Yellow arrows show inorganic filler agglomerates, red arrows indicate P(3HO) agglomerates, while green arrows exhibit cracks and areas of partial degradation of P(3HO).*

Table S3. *Young modulus (E) of the prepared nanocomposites. Data are presented as mean ± SE.*

**Sample abbreviations**	***E* (MPa)**
**P(3HO)**	11.1 ± 0.8
**P(3HO)_ZnO NPs**	14.0 ± 2.0
**P(3HO)_Zn/Al-turm**	8.2 ± 2.2
**P(3HO)_Ca/Al**	8.7 ± 1.2
**P(3HO)_Ca/Al-turm**	9.1 ± 0.2

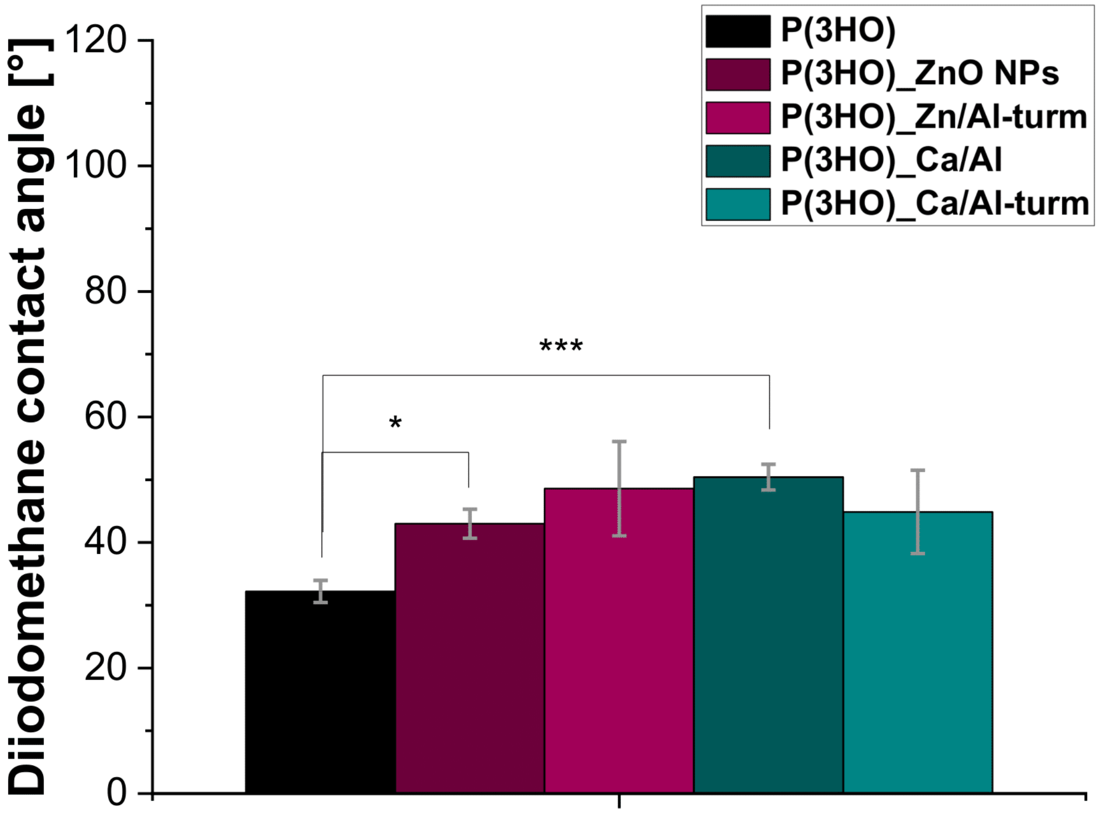

Figure S7. *The wettability of the materials treated with diiodomethane (n = 6; error bars = ±SD). Results are statistically significant where: *p < 0.05, ***p < 0.001.*

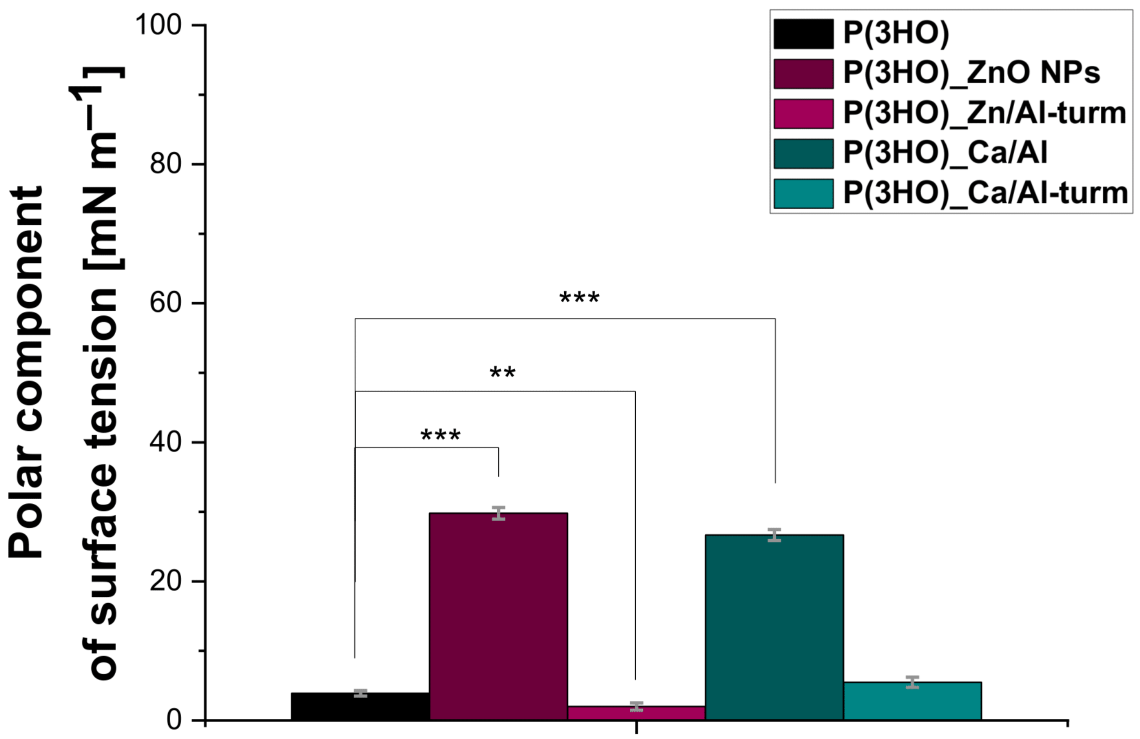

Figure S8. *The polar component of the surface tension (n = 36; error bars = ±SD). Results are statistically significant where: **p < 0.01, ***p < 0.001.*

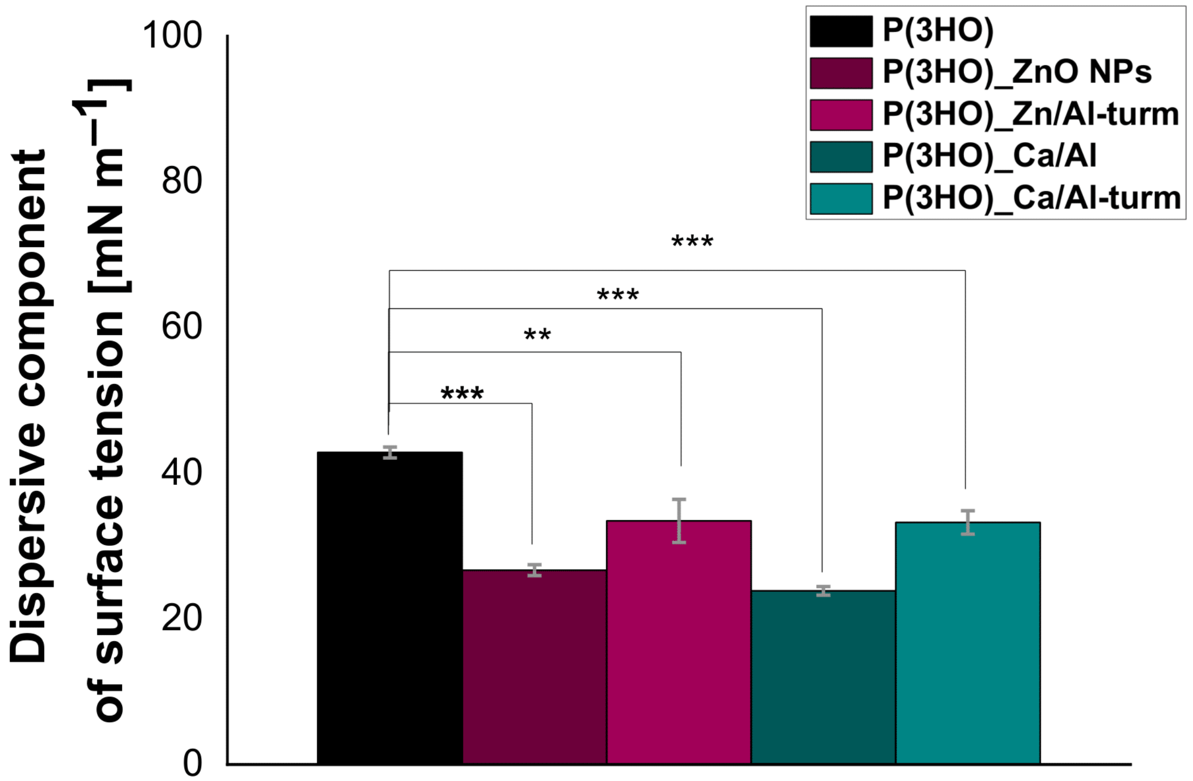

Figure S9. *The dispersive component of the surface tension (n = 36; error bars = ±SD). Results are statistically significant where: **p < 0.01, ***p < 0.001.*

Table S4. *The wettability of the nanocomposites treated with water and diiodomethane. Data are presented as mean ± SE.*

**Sample abbreviations**	**Water [°]**	**Diiodomethane [°]**
**P(3HO)**	79.5 ± 1.6	32.2 ± 1.8
**P(3HO)_ZnO NPs**	78.7 ± 0.8	43.0 ± 2.3
**P(3HO)_Zn/Al-turm**	92.8 ± 0.8	48.6 ± 7.5
**P(3HO)_Ca/Al**	84.5 ± 5.2	50.4 ± 2.0
**P(3HO)_Ca/Al-turm**	82.2 ± 5.2	44.9 ± 6.6

Table S5. *The dispersive ($\boldsymbol{\gamma_S^P}$) and polar components ($\boldsymbol{\gamma_S^P}$), as well as the surface tension ($\boldsymbol{\gamma_S}$) of the nanocomposites. Data are presented as mean ± SE.*

**Sample abbreviations**	γ_S^P **[mN m⁻¹]**	γ_S^P **[mN m⁻¹]**	γ_S **[mN m⁻¹]**
**P(3HO)**	42.8 ± 0.8	3.9 ± 0.4	46.7 ± 0.7
**P(3HO)_ZnO NPs**	26.7 ± 0.7	29.8 ± 0.8	56.5 ± 0.8
**P(3HO)_Zn/Al-turm**	33.4 ± 2.9	2.0 ± 0.5	35.4 ± 2.5
**P(3HO)_Ca/Al**	23.8 ± 0.6	26.7 ± 0.8	50.5 ± 0.8
**P(3HO)_Ca/Al-turm**	33.2 ± 1.6	5.5 ± 0.7	38.7 ± 1.4

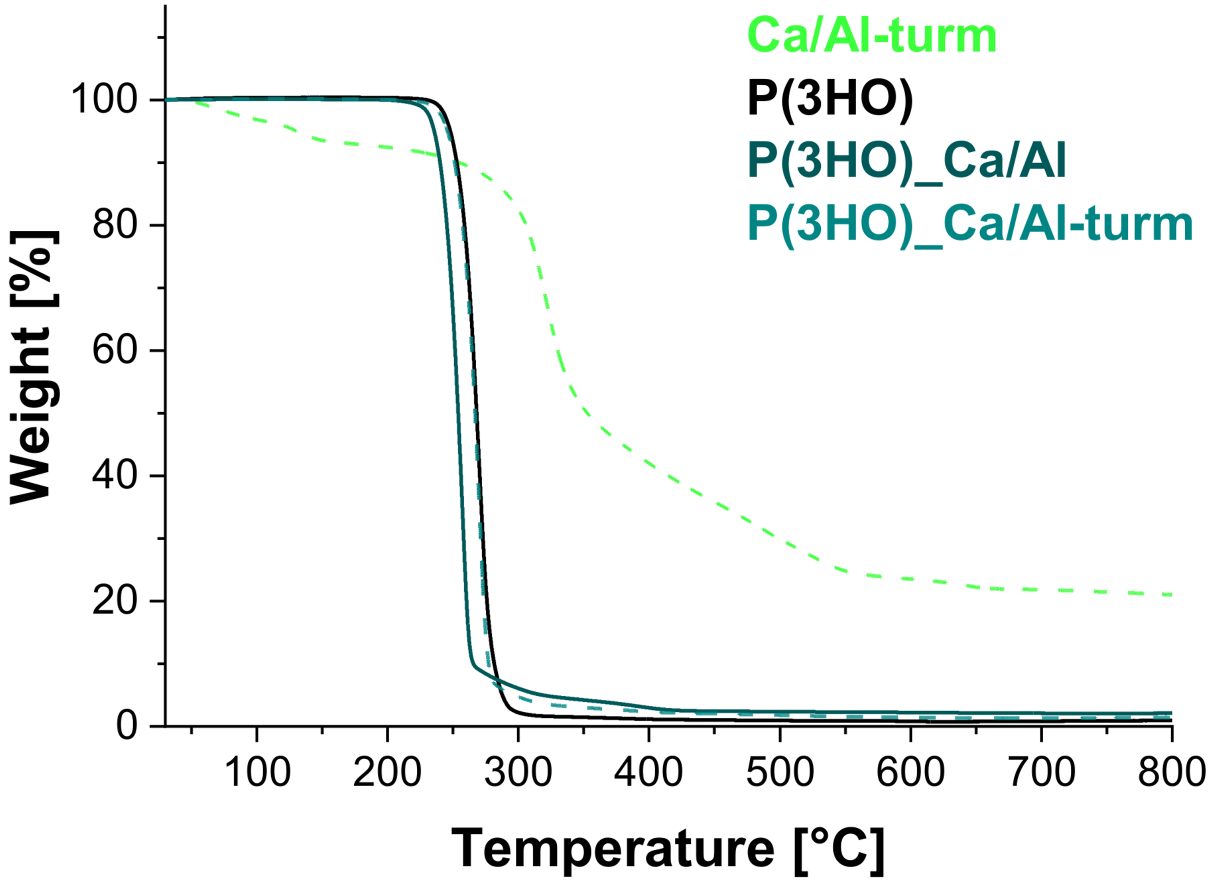

Figure S10. *TGA curves of neat P(3HO), Ca/Al-turm, and P(3HO)-based nanocomposites containing Ca/Al (P(3HO)_Ca/Al), Ca/Al-turm (P(3HO)_Ca/Al-turm).*

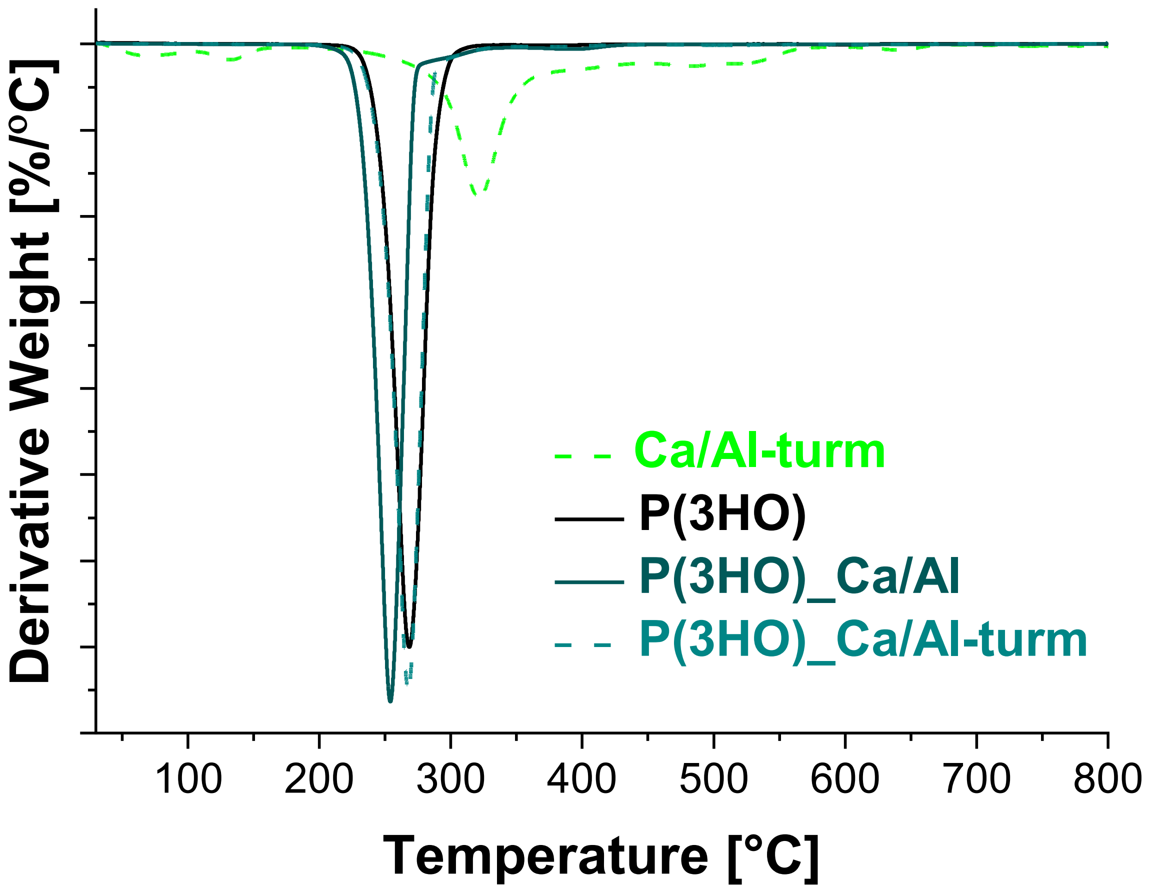

Figure S11. *DTG curves of neat P(3HO), Ca/Al-turm, and P(3HO)-based nanocomposites containing Ca/Al (P(3HO)_Ca/Al), Ca/Al-turm (P(3HO)_Ca/Al-turm).*

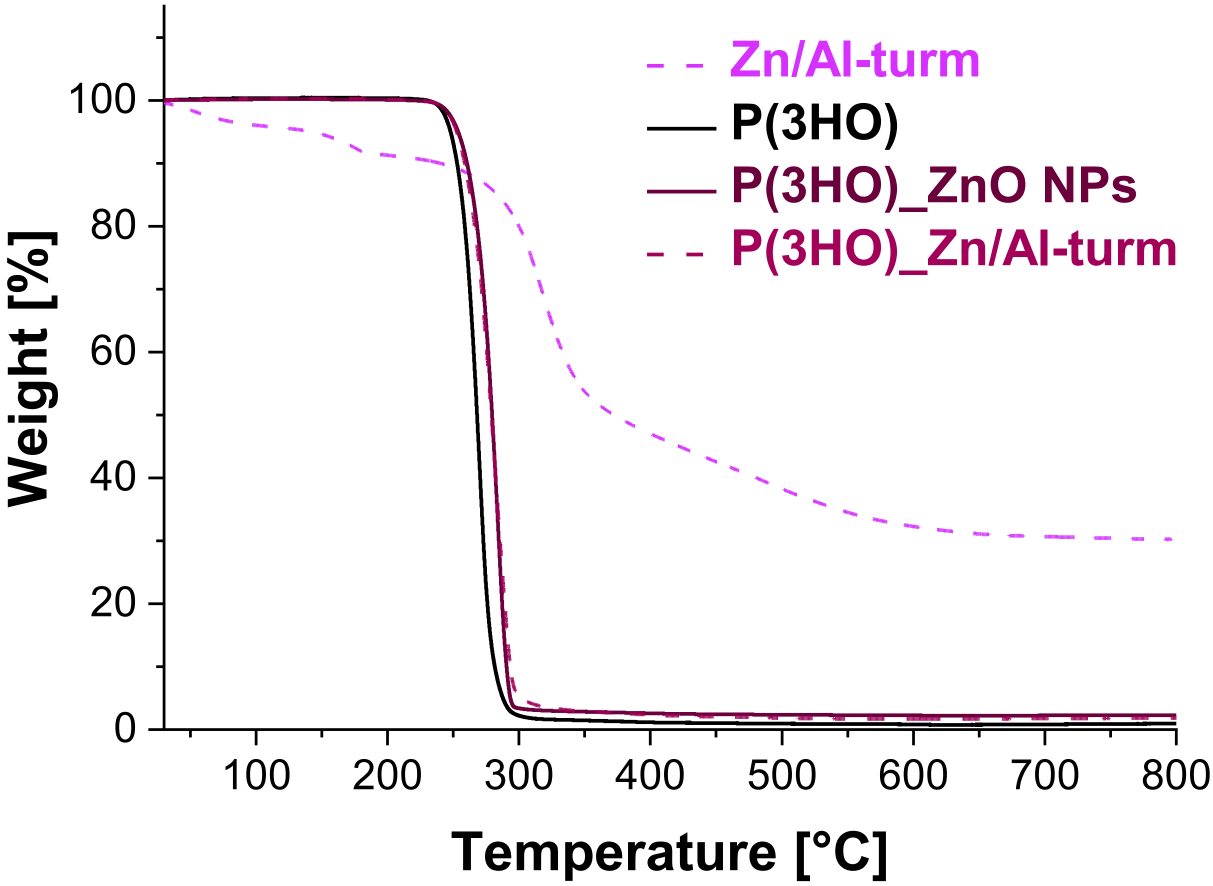

(3HO)

Figure S12. *TGA curves of neat P(3HO), Zn/Al-turm, and P(3HO)-based nanocomposites containing ZnO NPs (P(3HO)_ZnO NPs), Zn/Al-turm (P(3HO)_Zn/Al-turm). TGA curves of P(3HO)_ZnO NPs and ZnO NPs were presented in our previous works*[7,8]*.*

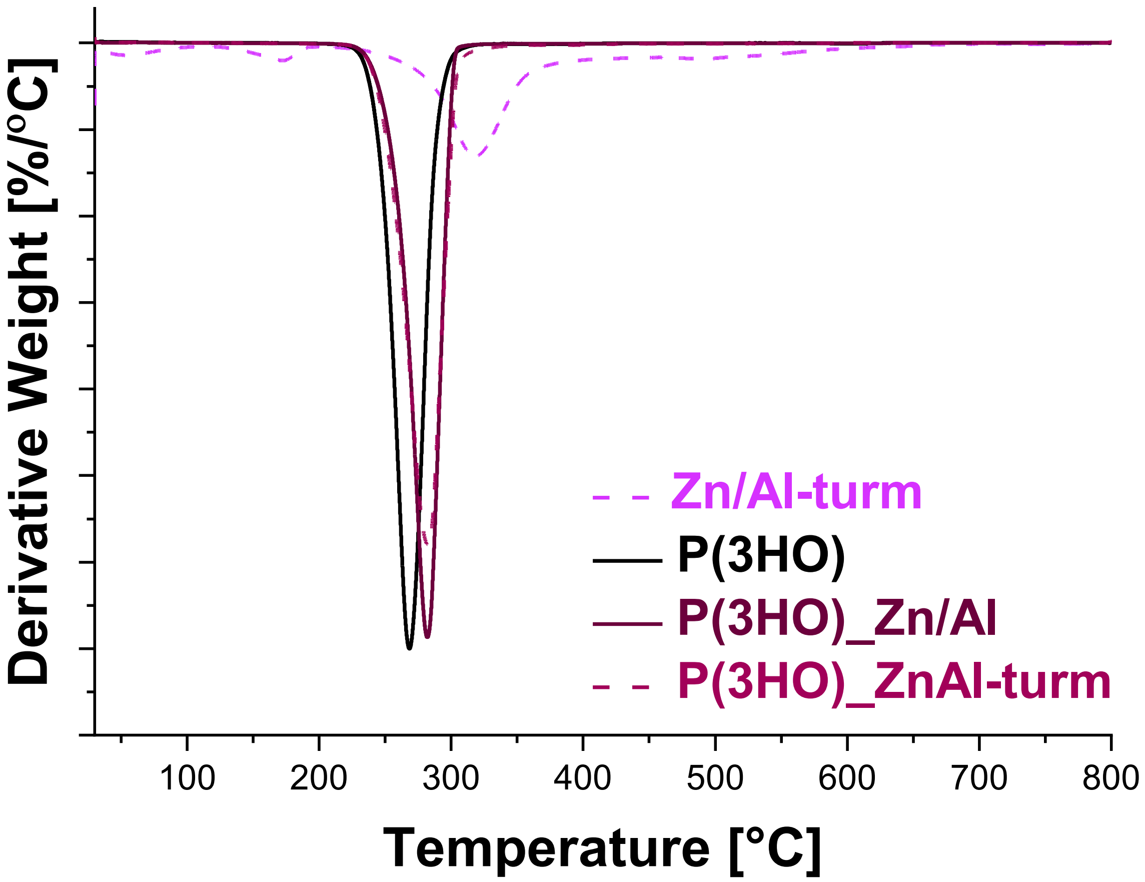

Figure S13. *DTG curves of neat P(3HO)*, Zn/*Al-turm*, *and P(3HO)*-based *nanocomposites containing* ZnO NPs *(P(3HO)_*ZnO NPs*)*, Zn/*Al-turm (P(3HO)_*Zn/*Al-turm*). *DTG curves of P(3HO)_ZnO NPs and ZnO NPs were presented in our previous works*[7,8].

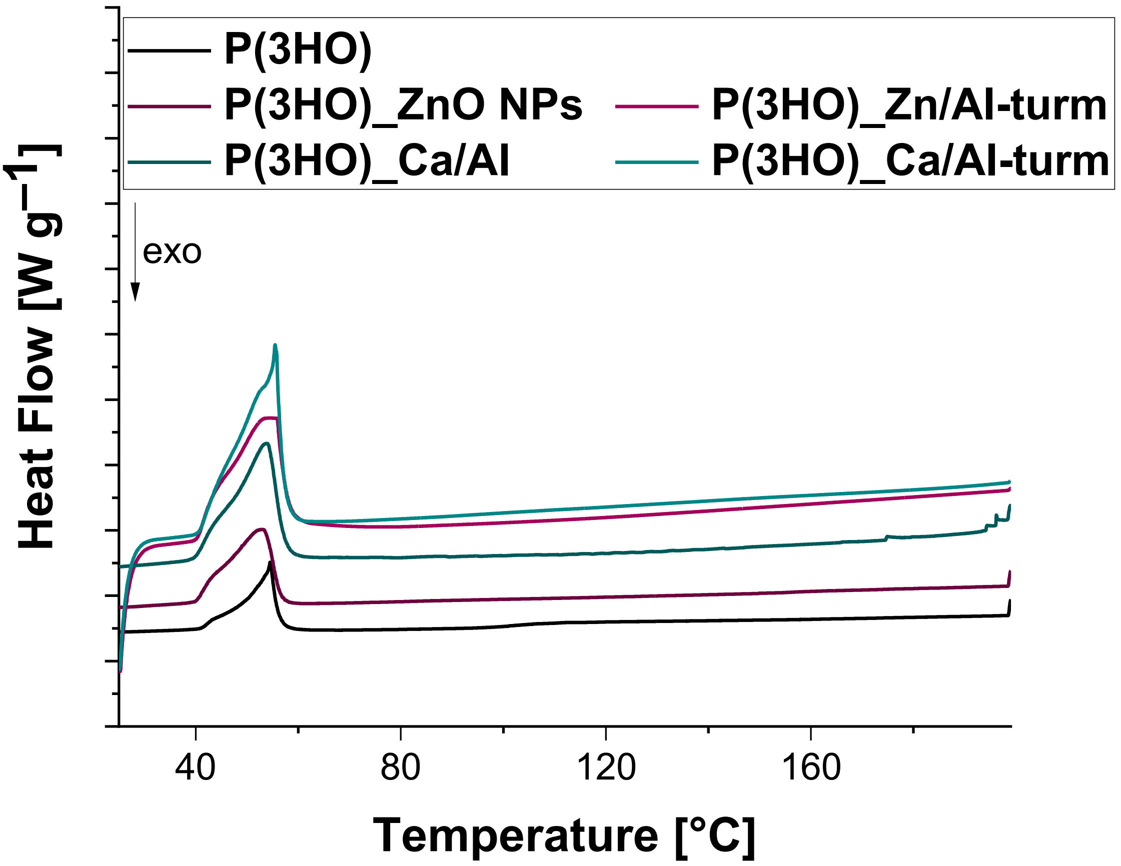

Figure S14. *DSC curves (first heating) of P(3HO)-based nanocomposites. DSC analysis of P(3HO), P(3HO)_ZnO NPs and P(3HO)_Ca/Al were presented in our previous work*[7]

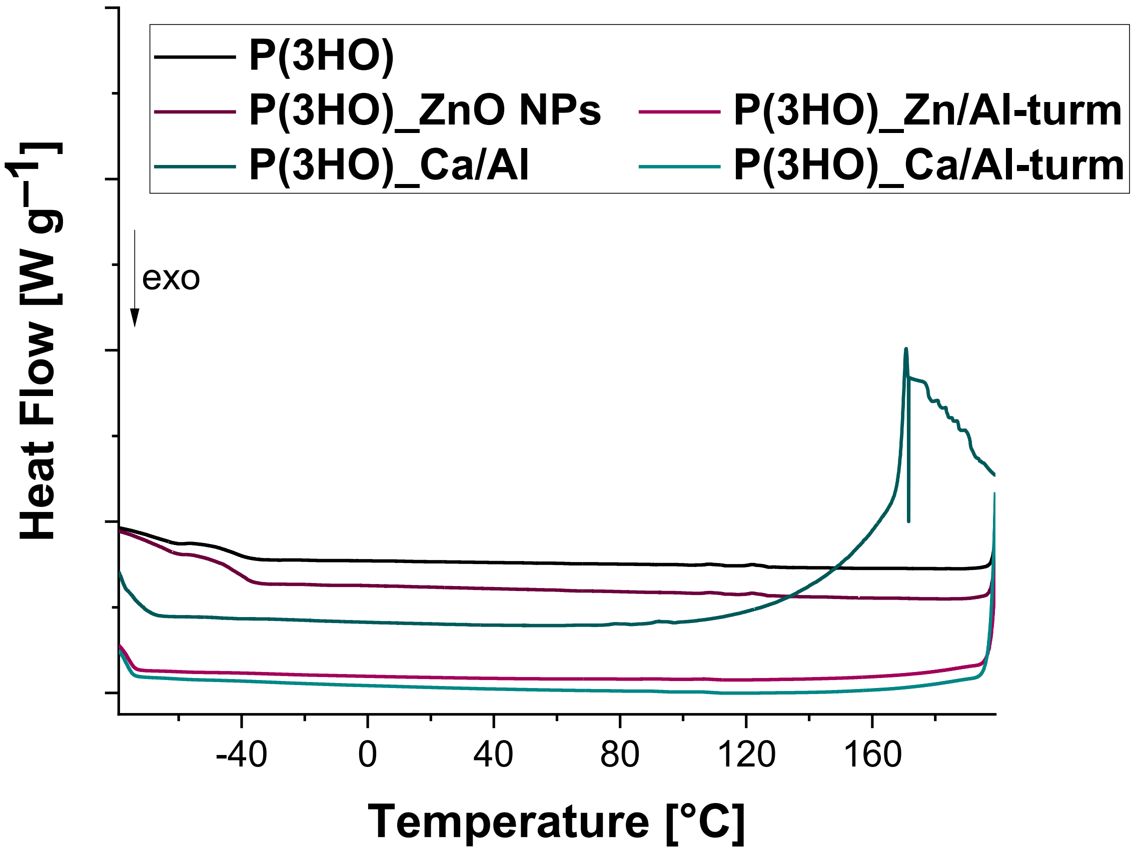

Figure S15. *DSC curves (cooling) of P(3HO)-based nanocomposites. DSC analysis of P(3HO), P(3HO)_ZnO NPs and P(3HO)_Ca/Al were presented in our previous work*[7]

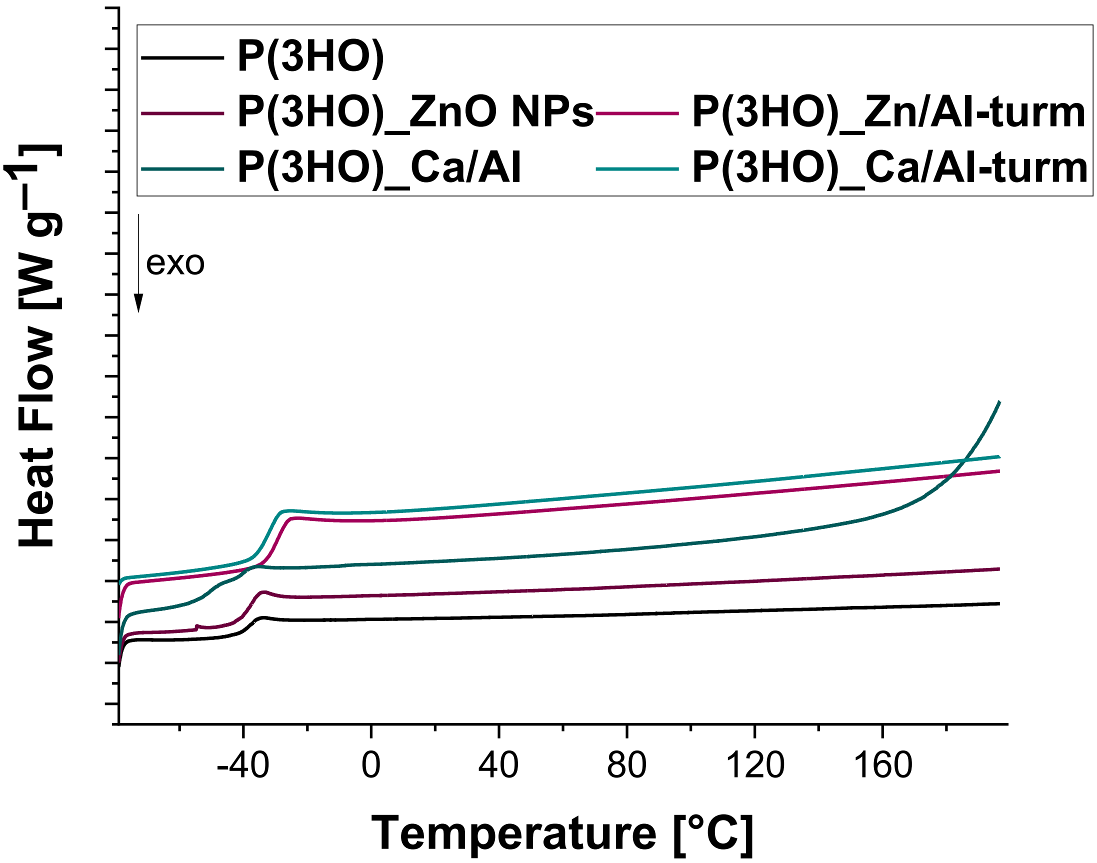

Figure S16. *DSC curves (second heating) of P(3HO)-based nanocomposites. DSC analysis of P(3HO), P(3HO)_ZnO NPs and P(3HO)_Ca/Al were presented in our previous work*[7]

Table S6. *Indirect cytotoxicity of the prepared nanocomposites. Data are presented as mean ± SE.*

Sample abbreviations	MC3T3-E1 [%]	NG108-15 [%]
**Negative control**	100.0 ± 4.0	100.0 ± 2.6
**P(3HO)**	103.6 ± 2.6	92.4 ± 0.5
**P(3HO)_ZnO NPs**	94.8 ± 2.1	99.7 ± 0.6
**P(3HO)_Zn/Al-turm**	78.8 ± 6.5	98.2 ± 0.6
**P(3HO)_Ca/Al**	104.0 ± 1.5	99.1 ± 1.3
**P(3HO)_Ca/Al-turm**	85.6 ± 7.0	101.0 ± 1.6

Table S7. *Direct cytotoxicity of the prepared nanocomposites. Data are presented as mean ± SE.*

Sample abbreviations	MC3T3-E1 [%]	NG108-15 [%]
**Negative control**	100.0 ± 2.4	100.0 ± 2.1
**P(3HO)**	93.6 ± 1.1	94.4 ± 1.3
**P(3HO)_ZnO NPs**	89.6 ± 1.4	91.0 ± 2.2
**P(3HO)_Zn/Al-turm**	89.2 ± 1.8	97.3 ± 0.9
**P(3HO)_Ca/Al**	99.4 ± 1.1	92.2 ± 0.7
**P(3HO)_Ca/Al-turm**	82.7 ± 2.3	92.2 ± 0.7

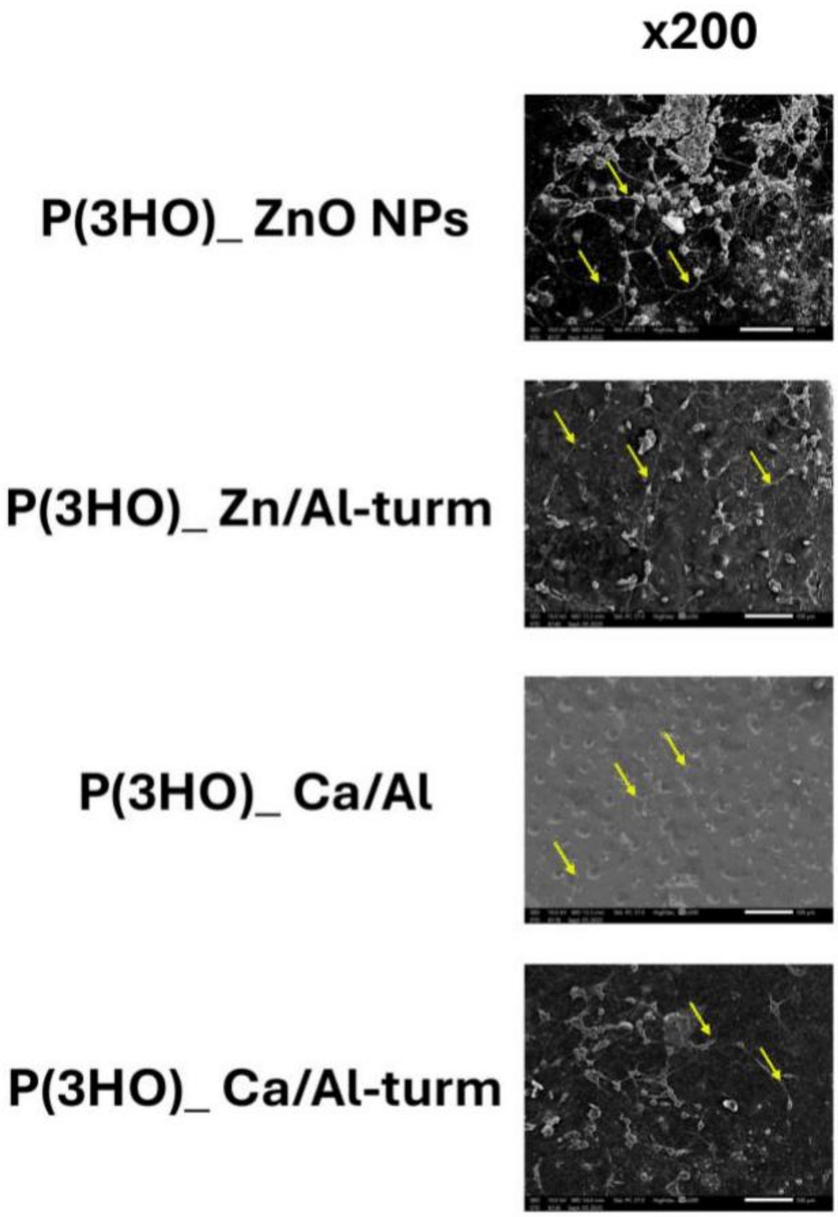

Figure S17. *Surface morphology of the nanocomposites on day 7 of the proliferation assay. Yellow arrows mark NG108-15 cells after differentiation into neurons.*

Table S8. *Quantification of TGF-β1 and MMP-2 in Culture Supernatants of PMA-Differentiated THP-1 Macrophages after 10th days of experiments. Data are presented as mean ± SE.*

**Sample abbreviations**	**TGF-β1 [pg mL^{-1}]**	**MMP-2 [pg mL^{-1}]**
**M0**	301.3 ± 21.1	24.3 ± 0.9
**M1**	325.1 ± 29.3	19.7 ± 1.8
**M2**	1787.2 ± 166.2	58.8 ± 17.8
**P(3HO)**	344.1 ± 15.7	23.7 ± 6.0
**P(3HO)_ZnO NPs**	307.7 ± 15.4	45.4 ± 11.8
**P(3HO)_Zn/Al-turm**	1787.2 ± 151.1	19.9 ± 1.6
**P(3HO)_Ca/Al**	650.3 ± 231.4	23.0 ± 11.1
**P(3HO)_Ca/Al-turm**	818.0 ± 140.6	34.8 ± 4.8

Table S9. *Composition of Simulated Body Fluid (SBF) and Ringer's fluid. Data are presented as mean ± SE.*

Component	SBF Solution	Ringer's fluid
**NaCl [g L^{-1}]**	8.035	8.600
**$NaHCO_3$ [g L^{-1}]**	0.355	-
**KCl [g L^{-1}]**	0.225	0.300
**$K_2HPO_4 \cdot 3H_2O$ [g L^{-1}]**	0.231	-
**$MgCl_2 \cdot 6H_2O$ [g L^{-1}]**	0.311	-
**1M HCl [mL L^{-1}]**	40.000	-
**$CaCl_2$ [g L^{-1}]**	0.292	0.243
**Na_2SO_4 [g L^{-1}]**	0.072	-
**Tris* [g L^{-1}]**	6.118	-

* *TRIS IUPAC name: 2-Amino-2-hydroxymethyl-propane-1,3-diol*

Literature:

(1) Wang, M.; Ren, F.; Zhou, J.; Cai, G.; Cai, L.; Hu, Y.; Wang, D.; Liu, Y.; Guo, L.; Shen, S. N Doping to ZnO Nanorods for Photoelectrochemical Water Splitting under Visible Light: Engineered Impurity Distribution and Terraced Band Structure. *Sci. Rep.* **2015**, *5*. https://doi.org/10.1038/srep12925.

(2) Kane, A. O.; Ngom, B. D.; Sakho, O. Influence of Adansonia Digitata Leaves Dye Extraction Solvent Nature on the Structural and Physical Properties of Biosynthesized ZnO Nanoparticles. In *Materials Today: Proceedings*; Elsevier Ltd, 2019; Vol. 36, pp 290–297. https://doi.org/10.1016/j.matpr.2020.04.048.

(3) Paulo, L.; Benício, F.; Eulálio, D.; Moura, L. De; Garcia, F. Layered Double Hydroxides as Hosting Matrices for Storage and Slow Release of Phosphate Analyzed by Stirred-Flow Method 2 . Materials and Methods. **2018**, *21* (3).

(4) Wu, S.; Liang, H.; Xu, B.; Zhang, Q.; Fan, H.; Wang, J.; Han, Q.; Gao, M. A Co - Precipitation Route for the Preparation of Eco - Friendly Cu - Al - Layered Double Hydroxides with Efficient Tetracycline Degradation. **2023**, 99412–99426. https://doi.org/10.1007/s11356-023-29345-4.

(5) López-tobar, E.; Blanch, G. P.; Ruiz, M. L.; Sanchez-cortes, S. Vibrational Spectroscopy Encapsulation and Isomerization of Curcumin with Cyclodextrins Characterized by Electronic and Vibrational Spectroscopy. *Vib. Spectrosc.* **2012**, *62*, 292–298. https://doi.org/10.1016/j.vibspec.2012.06.008.

(6) Luisa, M.; López-tobar, E.; Sanchez-cortes, S.; Flores, G.; Patricia, G. Vibrational Spectroscopy Stabilization of Curcumin against Photodegradation by Encapsulation in Gamma-Cyclodextrin : A Study Based on Chromatographic and Spectroscopic (Raman and UV – Visible) Data. *Vib. Spectrosc.* **2015**, *81*, 106–111. https://doi.org/10.1016/j.vibspec.2015.10.008.

(7) Haraźna, K.; Grzela, K.; Stępień-Hołubczat, K.; Beneš, H.; Hodan, J.; Nevoralová, M.; Medeiros, G. S.; Bujok, S.; Guzik, M. Novel, Eco-Friendly α-Tocopherol/Layered Double Hydroxides/Poly(3-Hydroxyoctanoate)-Based Active Packaging Materials with Enhanced Antimicrobial, Barrier and Antioxidant Activities. *Chemical Engineering Journal* **2024**, *500*. https://doi.org/10.1016/j.cej.2024.156959.

(8) Bujok, S.; Peter, J.; Halecký, M.; Ecorchard, P.; Machálková, A.; Santos Medeiros, G.; Hodan, J.; Pavlova, E.; Beneš, H. Sustainable Microwave Synthesis of Biodegradable Active Packaging Films Based on Polycaprolactone and Layered ZnO Nanoparticles. *Polym. Degrad. Stab.* **2021**, *190*. https://doi.org/10.1016/j.polymdegradstab.2021.109625.
